# Recurrent homozygous deletion of *DROSHA* and microduplication of *PDE4DIP* in pineoblastoma

**DOI:** 10.1038/s41467-018-05029-3

**Published:** 2018-07-20

**Authors:** Matija Snuderl, Kasthuri Kannan, Elke Pfaff, Shiyang Wang, James M. Stafford, Jonathan Serrano, Adriana Heguy, Karina Ray, Arline Faustin, Olga Aminova, Igor Dolgalev, Stacie L. Stapleton, David Zagzag, Luis Chiriboga, Sharon L. Gardner, Jeffrey H. Wisoff, John G. Golfinos, David Capper, Volker Hovestadt, Marc K. Rosenblum, Dimitris G. Placantonakis, Sarah E. LeBoeuf, Thales Y. Papagiannakopoulos, Lukas Chavez, Sama Ahsan, Charles G. Eberhart, Stefan M. Pfister, David T. W. Jones, Matthias A. Karajannis

**Affiliations:** 1 Division of Neuropathology, NYU Langone Health, New York, 10016 NY USA; 2 Laura and Isaac Perlmutter Cancer Center, NYU Langone Health, New York, 10016 NY USA; 3 Department of Neurology, NYU Langone Health, New York, 10016 NY USA; 4 Department of Pathology, NYU Langone Health, New York, 10016 NY USA; 5 Hopp Children’s Cancer Center at the NCT Heidelberg (KiTZ), Heidelberg, 69120 Germany; 6 Division of Pediatric Neurooncology, German Cancer Research Center (DKFZ), Heidelberg, 69120 Germany; 7 Department of Pediatric Oncology, Hematology & Immunology, Heidelberg, University Hospital, Heidelberg, 69120 Germany; 8 Division of Pediatric Hematology/Oncology, NYU Langone Health, New York, 10016 NY USA; 9 Department of Biochemistry and Molecular Pharmacology, NYU Langone Health, New York, 10016 NY USA; 10 Genome Technology Center, NYU Langone Health, New York, 10016 NY USA; 11 Johns Hopkins All Children’s Hospital, Cancer and Blood Disorders Institute, St Petersburg, 33701 FL USA; 12 Department of Neurosurgery, NYU Langone Health, New York, 10016 NY USA; 13 Division of Pediatric Neurosurgery, NYU Langone Health, New York, 10016 NY USA; 14 Department of Neuropathology, Institute of Pathology, University of Heidelberg, Heidelberg, 69120 Germany; 15 Clinical Cooperation Unit Neuropathology, German Cancer Consortium (DKTK), German Cancer Research Center (DKFZ), Heidelberg, 69120 Germany; 16 Division of Molecular Genetics, German Cancer Research Center (DKFZ), Heidelberg, 69120 Germany; 17 Department of Pathology, Memorial Sloan Kettering Cancer Center, New York, 10065 NY USA; 18 Neuroscience Institute and Kimmel Center for Stem Cell Biology, NYU Langone Health, New York, 10016 NY USA; 19 Division of Pediatric Oncology, Johns Hopkins Hospital, Baltimore, 21218 MD USA; 20 Department of Pathology, Johns Hopkins Hospital, Baltimore, 21218 MD USA; 21 Department of Pediatrics, Memorial Sloan Kettering Cancer Center, New York, 10065 NY USA; 22 Present Address: Charité — Universitätsmedizin Berlin, corporate member of Freie Universität Berlin, Humboldt-Universität zu Berlin, and Berlin Institute of Health, Department of Neuropathology, Berlin, 10117 Germany

## Abstract

Pineoblastoma is a rare and highly aggressive brain cancer of childhood, histologically belonging to the spectrum of primitive neuroectodermal tumors. Patients with germline mutations in *DICER1*, a ribonuclease involved in microRNA processing, have increased risk of pineoblastoma, but genetic drivers of sporadic pineoblastoma remain unknown. Here, we analyzed pediatric and adult pineoblastoma samples (*n* = 23) using a combination of genome-wide DNA methylation profiling and whole-exome sequencing or whole-genome sequencing. Pediatric and adult pineoblastomas showed distinct methylation profiles, the latter clustering with lower-grade pineal tumors and normal pineal gland. Recurrent variants were found in genes involved in PKA- and NF-κB signaling, as well as in chromatin remodeling genes. We identified recurrent homozygous deletions of *DROSHA*, acting upstream of *DICER1* in microRNA processing, and a novel microduplication involving chromosomal region 1q21 containing *PDE4DIP* (myomegalin), comprising the ancient DUF1220 protein domain. Expresion of PDE4DIP and DUF1220 proteins was present exclusively in pineoblastoma with *PDE4DIP* gain.

## Abstract (web summary)

Pineoblastoma is a highly aggressive and rare childhood brain cancer, and the genetic drivers of sporadic pineoblastoma are unknown. Here, the authors genomically interrogated pediatric and adult pineoblastomas and found novel variants including recurrent homozygous deletions of *DROSHA*.

## Introduction

According to the 2016 World Health Organization (WHO) Classification of Tumors of the Central Nervous System, tumors of the pineal region comprise pineocytoma (grade I), pineal parenchymal tumor of intermediate differentiation (PPTID) (grade II or III), pineoblastoma (PB) (grade IV), and papillary tumor of the pineal region (grade II or III)^1–3^. PBs are very rare tumors, accounting for less than 1% of pediatric brain tumors, and our current knowledge of the molecular genetics and biology of PB is limited.

Histologically, PB closely resembles other “small round blue cell” embryonal tumors or primitive neuroectodermal tumors (PNET), such as medulloblastoma and retinoblastoma in the brain, and Ewing sarcoma/peripheral PNET, and neuroblastoma in the bone and adrenal gland, respectively. While the majority of PB appear to arise sporadically, known predispositions include germline mutations in the *RB1* and *DICER1* genes^1,2^. A subset of PBs arise in the setting of familial bilateral retinoblastoma, which is then termed trilateral retinoblastoma syndrome^3^. PBs have also been reported in patients with familial adenomatous polyposis^3^.

Genetic drivers of sporadic PBs are unknown. Despite morphological similarities, PBs do not seem to share any of the recurrent genetic drivers identified in medulloblastoma or in “CNS-PNET”^4^. Previous studies in PB have identified recurrent losses of chromosome 22q, as well as high level gains of 1q12, 5p13, 5q21, 6p12, and 14q21^1^, and microarray studies showed significant transcriptional upregulation of UBEC2, SOX4, TERT, TEP1, PRAME, CD24, POU4F2, and HOXD13^5,6^. In this study we sought to identify molecular drivers of sporadic PB to gain insights into the biology of this rare disease.

Here, we show that adult and pediatric PBs have different methylation profiles and harbor recurrent homozygous deletions of *DROSHA*, as well as microduplication of *PDE4DIP* containing the ancient DUF1220 domain, representing novel molecular drivers of PB. Furthermore, heterogeneity within the pediatric PB methylation cluster indicates the presence of biological subgroups within pediatric PB. Larger retrospective and prospective studies will be necessary to further delineate the diagnostic, prognostic, and therapeutic relevance of our findings for children afflicted with this rare cancer.

## Results and Discussion

### Genome-wide DNA methylation profiling

**Fig. 1:**
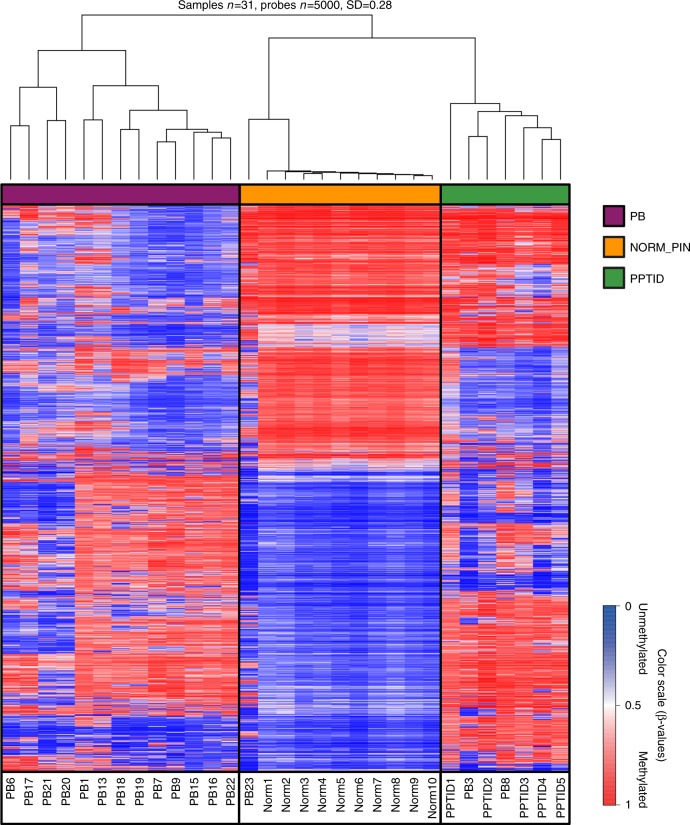
Methylation signature of pineoblastoma, pineal gland, and PPTID. Pediatric pineoblastomas form a distinct cluster that is separated from normal pineal gland and pineal parenchymal tumor of intermediate differentiation (PPTID). However, adult pineoblastomas cluster with PPTID (PB3 and PB8) as well as the normal pineal gland group (PB23), although analyzed tissues were confirmed as pineoblastoma, suggesting that methylation signature reflects origin of adult pineoblastomas from a normal pineal gland or a lower grade precursor lesion

To ascertain whether PBs and PPTID can be distinguished using genome-wide DNA methylation profiling, as shown for other pediatric brain tumors^4,7^; we performed unsupervised hierarchical clustering selecting the 5000 probes that showed the highest standard deviation (SD) across our samples. We compared 10 samples from normal autopsy pineal glands (average age 61 years), 16 tumors clinically and histologically diagnosed as PB and five tumors diagnosed as PPTID. Of 16 PBs, 13 formed a distinct cluster, while two tumors clustered with PPTID (PB3 and PB8) and one tumor (PB23) clustered closer to normal pineal tissue (Fig. 1). All three tumors were histologically and clinically confirmed as PBs. Strikingly, review of the clinical information showed that all three tumors were PB that arose in adults. Sample PB23, which clustered closely with the normal pineal gland group, albeit with noticeable differences (Fig. 1), was from a 69-year-old patient, the oldest in our group (Supplementary Fig. 1). Patients with PBs clustering with the PPTID samples were 45 (PB3) and 37 (PB8) years old at the time of diagnosis. This may suggest that PBs presenting in adults may arise from preexisting PPTIDs or normal pineal gland (secondary PB), whereas pediatric PBs might arise directly from embryonal neuroblasts (primary PB). However, this needs to be confirmed in larger studies, including comparison with high-grade PPTID. An alternative explanation is that at least some of the PBs in adults may actually represent misclassified pineal parenchymal tumors of intermediate differentiation; however, the diagnosis of PB was rendered by four experienced board certified neuropathologists.

### Genome sequencing

**Fig. 2:**
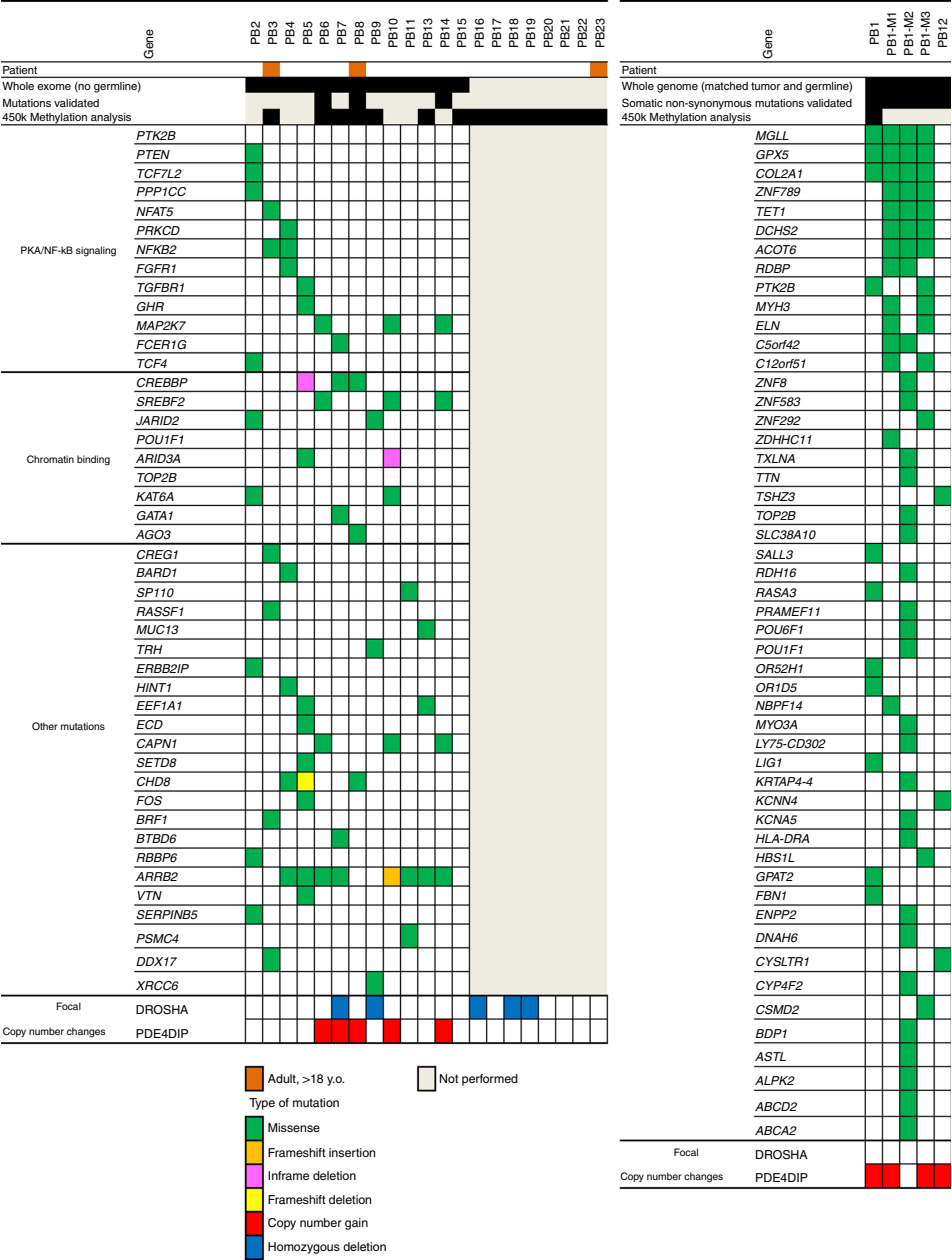
Genomic landscape of pineoblastoma. Whole-exome sequencing was performed in 13 tumors with no matching germline (exome samples, left). Whole-genome sequencing (genome samples, right) was performed in five tumors, two primary (PB1 and PB12) with matched peripheral blood DNA and three metastasis from PB1 (M1, M2, M3). Specific genes are shown that bear nonsynonymous mutations. In exome samples only mutations enriched in pathway analysis via Ingenuity Pathway Analysis (IPA) software are included. In genome samples, only somatic nonsynonymous mutations are presented. Sufficient material was available to confirm mutations in eight tumors (mutations validated). See also Supplementary Table 1 for mutation details

To identify candidate oncogenic drivers in PB, we performed whole-exome sequencing (WES) on 13 PB without available matching germline DNA and whole-genome sequencing (WGS) on five PB samples with matched normal germline DNA (Fig. 2, Table 1). Out of five WGS, two were primary PBs and three were synchronous metastases (M1–M3) from one of the WGS tumors (PB1) obtained after tumor recurrence during autopsy. Due to the small size of the samples, only eight tumors had sufficient DNA for validation of the identified mutations (Fig. 2). Mean tumor target coverage was 174× for WES and 33× for WGS. Mean normal target coverage for WGS was 36×. On average, we identified 1545 nonsynonymous putative mutations (WES) and 74 nonsynonymous somatic mutations in 51 genes (WGS) in exons. Similarly to other pediatric cancers, overall mutational burden in PB was relatively low (Supplementary Fig. 2), similar to other pediatric cancers.

**Table 1:** Overview of whole-genome (WGS) and whole-exome (WES) sequencing of pineoblastoma

Statistic	Whole exome	Whole genome
Tumor/tumor-normal pairs sequenced	13 (tumor only)	5 (2 primary + 3 mets) (tumor + matched germline)
Total Gb sequenced	144.1	15.0
Mean fold tumor target coverage	174.4	32.8
Mean fold normal target coverage	–	36.4
Mean number of exonic variants/mutations identified	1544.9	121.4
Mean number of nonsynonymous variants/mutations identified	522.3	83.6
Mean number of synonymous variants/mutations identified	795.2	31.8
Total number of validated nonsynonymous variants/mutations	137	74

In WGS samples, the primary tumor and metastatic foci shared somatic missense mutations in *MGLL*, *GPX5*, and *COL2A1*, while all three metastatic foci shared mutations in *ZNF789*, *TET1,* *DCHS2*, and *ACOT6*, which were not present in the primary tumor, suggesting these may be associated with the tumor progression or metastasis (Fig. 2). Interestingly, although PB1 and its metastases shared mutations, all three metastatic lesions showed a much higher mutation rate and had a large number of private mutations (Supplementary Fig. 2a, b**)**. Since the metastatic lesions were collected at the time of autopsy after radiation therapy and myeloablative high-dose chemotherapy, the increased mutational burden may represent at least in part the mutagenic effect of therapy, rather than a natural progression of the disease. Two primary PB, PB1 and PB12, did not share any somatic mutations. (Fig. 2). In WES samples, where only tumor tissue without matched germline DNA was available, we found enrichment by pathway analysis for mutations in the PKA/NFκB signaling pathway (*PTK2B, MAP2K7, CREBBP*), genes involved in chromatin binding (*SREBF2, AGO3, POU1F1, TOP2B*) and other pathways (*CAPN1, ARRB2, CHD8, SETD8*). The most commonly mutated gene in PBs was *ARRB2*, with mutations present in eight tumor samples with a hotspot in Arrestin_N domain (Supplementary Fig. 3). *MAP2K7*, *SREBF2*, and *CAPN1* mutations were present concurrently in three WES samples and appeared to cluster in hotspots (Fig. 2, Supplementary Fig. 3 and Supplementary Table 1). However, due to the unavailability of matching germline DNA from our samples, future studies are necessary to confirm the role of these mutations in PB. In one PB analyzed by WES (PB15), we could not identify any relevant mutations by pathway enrichment analysis (Fig. 2); however, the tumor showed aberrations in the copy-number profile affecting chromosomes 4, 8, 17, and 22 (Supplementary Fig. 4). Overall, copy-number profiles of pediatric PBs showed widespread chromosomal losses and gains, in contrast to PPTID and PBs of adults, which harbored few chromosomal gains or losses (Supplementary Fig. 4).

**Fig. 3:**
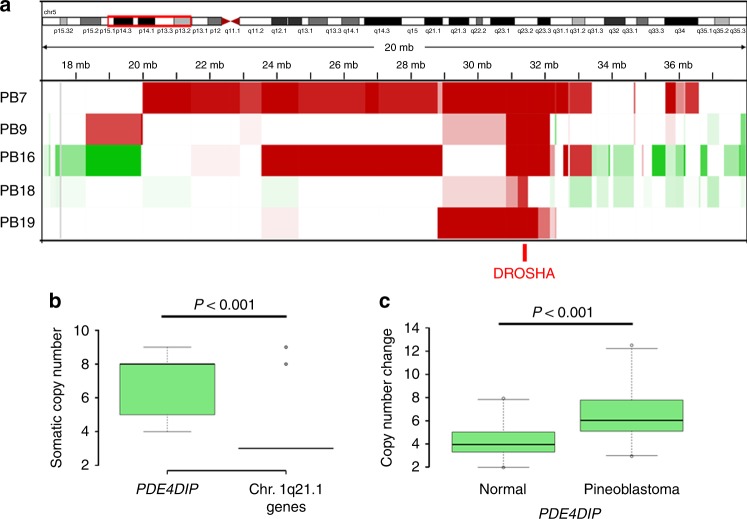
Focal copy-number aberrations in pineoblastoma. **a** Homozygous deletions of *DROSHA* were identified in five pediatric pineoblastomas. For four out of five cases with *DROSHA* deletion, the deletion is about 1.300–3.755 kb including the whole *DROSHA* gene. For one case, the deletion seems to be only 300 kb, spanning exons 11–35 of *DROSHA*. **b**, **c** We identified microduplication of *PDE4DIP* in seven pineoblastomas. **b** Whole-genome sequencing (*n* = 2) shows higher than expected copy number difference between *PDE4DIP* and neighboring genes in 1q21.1 region in pineoblastomas (*t*-test *p* value < 0.001). **c** Copy number of *PDE4DIP* in pineoblastoma is significantly increased compared to the number of *PDE4DIP* copies in normal DNA by ddPCR (average number of *PDE4DIP* copies in pineoblastoma six, vs. four in normal controls, paired *t*-test between normal and tumors <0.001). Genes neighboring *PDE4DIP* were not amplified by ddPCR confirming that the microduplication was limited to *PDE4DIP* only

Germline and somatic mutations in *DICER1* have been recognized as oncogenic drivers in PB^1^. Germline mutations in *DICER1* are associated with a distinct autosomal dominant tumor predisposition syndrome (OMIM #601200), whereby patients have an increased risk of developing a variety of extremely rare tumors, such as pleuropulmonary blastoma and PBs. However, *DICER1* is associated with susceptibility to PBs in only a small number of PBs. Interestingly, we did not observe any *DICER1* mutations in our samples despite excellent coverage by both WES and WGS (Supplementary Fig. 5). However, we identified homozygous deletions of *DROSHA* in 5 out of 19 samples (26%) (Figs. 2 and 3). *DROSHA* plays a critical role in miRNA processing in the nucleus, upstream of *DICER1*, therefore likely resulting in impaired miRNA biogenesis similarly to Wilms tumors, which carry both *DROSHA* and *DICER1* mutations^8,9^. In contrast to Wilms tumor, where *DROSHA* as well as *DICER1* are inactivated by hotspot mutations, we show that in PB *DROSHA*, similar to *DICER1*^1,10^, is inactivated by copy-number loss. Within the context of a relatively low mutation burden, this suggests that PB may be predominantly driven by recurrent copy-number alterations rather than mutations, although further genetic studies with paired tumor-normal are needed.

Further, using WGS, we identified copy-number gains of the gene coding for phosphodiesterase 4D interacting protein (*PDE4DIP*) in both primary tumor samples and two out of three metastases (M1 and M3) compared to neighboring 1q21 genes (Fig. 3b**)** Until recently, the latest human genome build (hg19) annotated a single copy of *PDE4DIP* per chromosome. Using a haploid human genome, O’Bleness et al.^11^ have since shown that the 1q21 region may contain up to 3 copies of *PDE4DIP*. Our five WGS tumor datasets showed eight copies of *PDE4DIP* on average, which would represent a microduplication. When we analyzed our WES samples by confirmatory droplet digital PCR (ddPCR) for *PDE4DIP*, the average *PDE4DIP* copy number was significantly higher in additional five PBs compared to normal pineal tissue controls (paired *t*-test, *p* < 0.001) (Fig. 3c). These gains were specific to *PDE4DIP*, and did not involve neighboring genes in the 1q21 region. In total, we identified gains of *PDE4DIP* in 7 out of 15 PBs for which sufficient DNA for confirmatory ddPCR was available. In cases where only DNA methylation was performed (PB16–23), *PDE4DIP* status could not be reliably confirmed due to the insufficient coverage by the 450k array in this region.

**Fig. 4:**
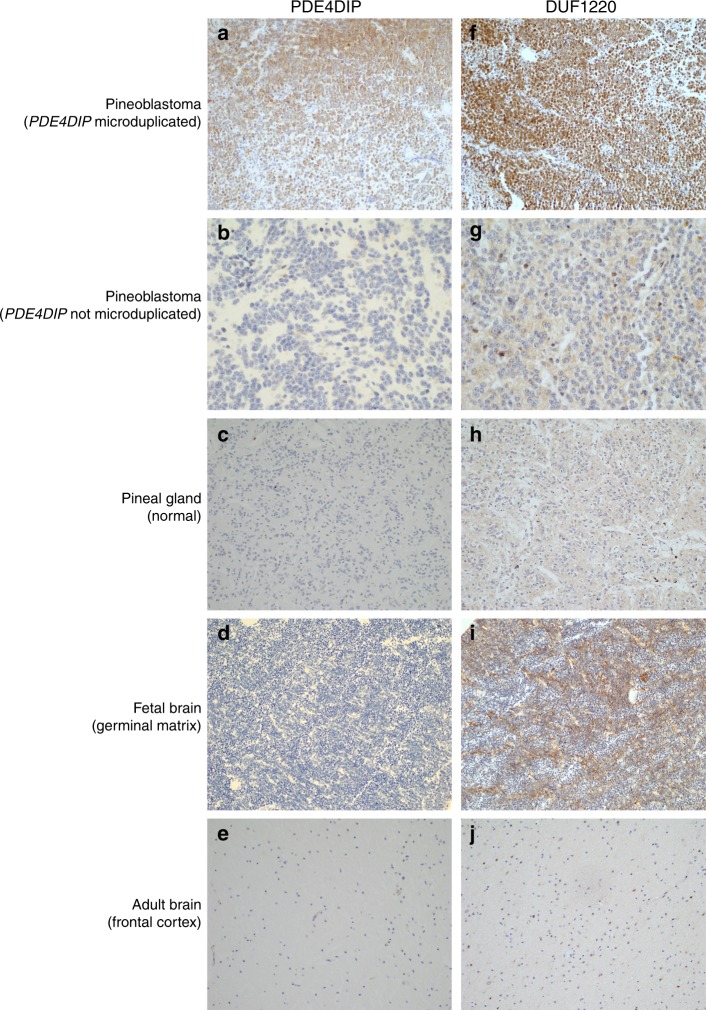
*PDE4DIP* microduplication correlates with overexpression of DUF1220. Pineoblastomas with *PDE4DIP* microduplication (*n* = 7) show strong expression of both PDE4DIP protein (**a**), as well as DF1220 protein (**f**), while pineoblastomas with no *PDE4DIP* microduplication (*n* = 5) (**b**, **g**) and normal pineal gland (**c**, **h**) are negative for both proteins. DUF1220 is strongly expressed in the germinal matrix of the fetal brain (**i**), consistent with its established role in human brain development; however, fetal brain neuroblasts are completely negative for PDE4DIP expression (**d**). Adult brain tissue is negative for both PDE4DIP and DUF1220 protein expression (**e**, **j**). (*n* = 5 for each normal control group, original magnification 400×, Scale bar 100 µm)

*PDE4DIP*, myomegalin, is a protein involved in cardiac muscle contraction and suggested to play a role in cardiomyopathy^12^. *PDE4DIP* is also among the genes affected by 1q21.1 microdeletion and 1q21.1 microduplication syndromes^13^. DUF1220 domains are interspersed throughout the 1q21 region, including within the *PDE4DIP* gene^11^. DUF1220 protein domains are approximately 65 amino acids in length and have undergone rapid and extensive copy-number expansion during mammalian evolution, and most strikingly in primate evolution including *Homo* species^14–16^. DUF1220 domains show the largest human-lineage-specific increase in copy number of any protein-coding region in the human genome. While humans carry ~289 copies of DUF1220, the copy number is significantly lower in other species, ranging from 125 in chimpanzees to 99 in gorillas, 4 in dolphins, 1 copy in mice, and 0 in non-mammals^11,14,15^. DUF1220 sequences exist within two distinct genomic environments. A single so-called ancestral domain is located in the *PDE4DIP* gene. The DUF1220 domain within *PDE4DIP* gene is present in primates, as well as other mammals. Other DUF1220 domains exist as multiple tandem copies in the *NBPF* genes, which are typically interspersed throughout the 1q21 region. Copy-number changes in DUF1220 appear to be directly linked to pathologic conditions affecting growth and function of the human brain. The number of DUF1220 copies in vertebrates also directly correlates with the number of cortical neurons^14^. Since DUF1220 plays a critical role in proliferation of neuroblasts during brain development and PB is a neuroectodermal tumor composed of primitive neuroblasts, we sought to establish whether the copy-number gain of *PDE4DIP* gene is associated with an increase of PDE4DIP and DUF1220 proteins. All PBs with *PDE4DIP* copy-number gain (*n* = 7) showed markedly increased expression of both PDE4DIP as well as DUF1220 proteins (Fig. 4), while normal pineal gland and adult brain were negative. Further evaluation of normal brain tissues revealed that neuroblasts in the germinal matrix of the fetal brain strongly express DUF1220, as would be expected from its presumed role in rapid neuroblastic proliferation; however, these neuroblasts did not express PDE4DIP protein. We did not observe overexpression of PDE4DIP or DUF1220 proteins in PBs without *PDE4DIP* microduplication (*n* = 5), confirming that genomic *PDE4DIP* gain is directly linked to the DUF1220 overexpression in PB (Fig. 4). Since the copy-number gain we detected in 1q21 was limited to *PDE4DIP* while neighboring regions showed a normal copy number, we concluded that the increase of DUF1220 protein in PBs is due to the copy-number gain of the ancient DUF1220 domain within *PDE4DIP*, and unlikely due to alterations in the *NBPF* gene group, where we did not detect any copy-number gains or mutations.

### Transcriptome analysis

**Fig. 5:**
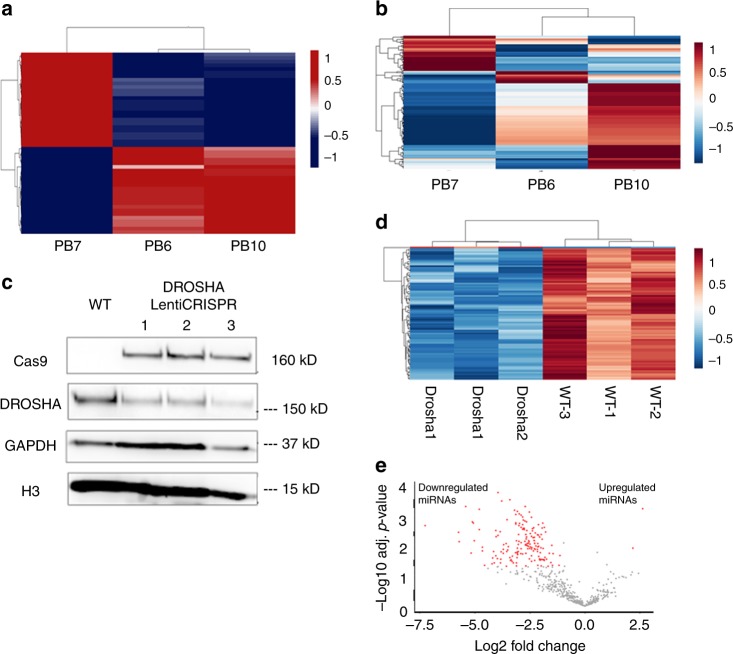
Effect of *DROSHA* alteration in pineoblastoma: Three human samples were available for RNA analysis. PB7 with concurrent homozygous *DROSHA* deletion and *PDE4DIP* gain, and PB6 and PB10 with *PDE4DIP* gain only. **a** Whole-transcriptome analysis shows that PB7 has distinctly different transcriptomic profile compared to PB6 and PB10 (unsupervised clustering, top 50 differentially up and down regulated genes shown), for detailed list see Supplementary Table 2. **b** PB7 with *DROSHA* deletion also shows markedly altered miRNA profile, (unsupervised clustering, top 200 differentially expressed miRNA shown, for detailed list see Supplementary Table 3). **c** Disruption of the *DROSHA* locus using CRISPR in human neural stem cells (hNSC) shows reduced levels of DROSHA protein, for quantification see Supplementary Fig. 6. **d, e** hNSC with mutation of *DROSHA* show markedly reduced levels of miRNA mirroring the clinical samples. **d** Unsupervised hierarchical clustering analysis based on expression data for the Top 85 differentially expressed miRNA show striking difference between *DROSHA*-mutated (Drosha1–3) and wild-type (WT1–3) hNSC samples. **e** Volcano plot shows marked reduction of miRNA in *DROSHA*-mutated hNSC. The *x* axis represents the difference of group means of DROSHA mutated and wild-type hNSC; the *y* axis represents the statistical significance. Each miRNa is represented by a dot, and red dots represent miRNAs that were significantly differentially expressed between groups. For detailed list of miRNAs see also Supplementary Table 4

To evaluate transcriptional differences between the effect of homozygous *DROSHA* deletion and *PDE4DIP* microduplication, we performed whole-transcriptome analysis of three PB cases where sufficient quantity and quality of RNA could be isolated from the formalin-fixed paraffin-embedded tissue (FFPE) samples. Two cases (PB6 and PB10) had gain of *PDE4DIP*, while one case (PB7) had concurrent gain of *PDE4DIP* and homozygous loss of *DROSHA*. Whole-transcriptome analysis showed that homozygous loss of *DROSHA* leads to distinct changes in RNA expression profile, compared to *PDE4DIP* gain only (Fig. 5a and Supplementary Table 2). We also performed miRNA profile analysis on clinical samples, confirming that the miRNA profile is markedly affected by loss of *DROSHA* (Fig. 5b and Supplementary Table 3). However, these analyses need to be confirmed in a larger cohort of PB samples from patients with different ages and underlying mutations. To evaluate the effect of *DROSHA* mutation experimentally, we disrupted the *DROSHA* locus in human neural stem cells (hNSC) using the CRISPR/Cas9 system, leading to decrease of the DROSHA protein, and massive loss of miRNAs in *DROSHA*-mutated hNSC cell lines (Fig. 5c-e, Supplementary Fig. 6 and Supplementary Table 4), consistent with previous observations of *DROSHA* and *DICER1* effect on miRNA^10,17^.

### Conclusions

In summary, we show that pediatric and adult PBs have distinct methylation profiles, suggesting that pediatric tumors arise de novo, whereas adult PBs may arise from a pineal parenchymal tumor or a normal pineal gland. We identified recurrent variants in several oncogenic signaling pathways including PKA, NFκB, and chromatin binding genes, as well as homozygous deletions of *DROSHA*, but no mutations in *DICER1*, suggesting that at least two different paths disrupting miRNA processing are involved in the pathogenesis of these tumors. Lastly, we identified a novel microduplication of *PDE4DIP* leading to upregulation of DUF1220 protein. The critical role of DUF1220 in proliferation of neuroblasts during normal embryonal brain development suggests DUF1220 as a novel oncogenic driver in PB.

## Methods

### Tissue collection

Tumor, normal pineal gland, and control brain tissues were obtained from the archives of the Departments of Neuropathology, New York University (NYU) Langone Medical Center and School of Medicine, and Johns Hopkins University. This study was approved by the Institutional Review Board of NYU Langone Medical Center (IRB #14-00948), and informed consent was obtained from study patients in accordance with all insititutional requirements. All specimens were reviewed and classified according to the World Health Organization (WHO) classification^3^ to confirm histological diagnosis and select blocks for DNA extraction and immunohistochemistry. Available clinical data are summarized in the Supplementary Table 5.

### DNA extraction

DNA was extracted from FFPE tissue. Areas with the highest available tumor content were chosen. Extraction was carried out using the automated Maxwell system (Promega, Madison, USA).

### Whole-exome and whole-genome sequencing and analysis

Exome sequencing: 250 ng of DNA from each sample were sheared on a Covaris instrument for 360 s (Duty cycle − 10%; intensity − 5; cycles/Burst − 200). Barcoded libraries were prepared using the Kapa Low-Throughput Library Preparation Kit Standard (Kapa Biosystems), amplified using the KAPA HiFi Library Amplification kit (Kapa Biosystems) (eight cycles) and quantified using Qubit Fluorimetric Quantitation (Invitrogen) and Agilent Bioanalyzer. An equimolar pool of the four barcoded libraries (300 ng each) was used as input to capture the exome using one reaction tube of the Nimblegen SeqCap EZ Human Exome Library v3.0 (Roche, cat # 06465684001), according to the manufacturer’s protocol. The pooled capture library was quantified by Qubit (Invitrogen) and Bioanalyzer (Agilent), and sequenced on an Illumina HiSeq 2500 using a paired end, 100 nucleotides in length run mode to achieve an average of 100× coverage.

Whole-genome sequencing: 500 ng of DNA from each sample were sheared on a Covaris instrument as described above, and barcoded libraries were prepared using the Kapa Low-Throughput Library Preparation Kit Standard (Kapa Biosystems), without any PCR amplification.

Fastq reads from both exome and genome sequencing were aligned to human genome build GRCh37 using BWA^18^ and recalibrated using GATK^19^. In the case of WGS, mutations were detected via Somatic Sniper^20^ and Somatic Indel Detector^19^ using matched normals. For the exomes we used VarScan^21^. All identified variants were annotated using Annovar^22^, and manually reviewed on IGV. Variants found in 1000 Genomes Project^23,24^, ESP6500, or dBSNP142^25^ were excluded, unless also documented in COSMIC^26^. Significance for pathway enrichment was calculated via IPA (Ingenuity Systems) Ingenuity Pathway Analysis software and ToppGene, with both corrected by the Benjamini Hochberg procedure. Identified mutations were validated on MiSeq platform, followed by manual IGV review in eight PBs with sufficient material available for confirmation. Variant allele frequencies and coverage for validated variants are listed in the Supplementary Table 6. CNV analysis was performed using Control-FREEC^27^, an algorithm for detection of copy-number changes and allelic imbalances (including LOH) using deep-sequencing data. Control-FREEC automatically computes, normalizes, segments copy number, and beta allele frequency (BAF) profiles, then calls copy-number alterations and LOH.

### Droplet digital PCR (ddPCR)

Droplet digital PCR was performed on a Bio-Rad QX200 to verify the CNV region of chromosome 1q22. Primers were designed against regions of amplification for *PDE4DIP* and *SEC22B* and unamplified regions of *HFE2* in the same chromosomal region. The RRP30 gene (diploid) was used as copy-number control, and normal pineal gland retrieved at autopsy as a normal tissue control. Primers will be provided upon request. A 20 ng of HindIII digested genomic DNA were used per reaction, using the following protocol: 1 cycle at 95 °C for 5 min, 40 cycles at 95 °C for 15 s, and 60 °C for 1 min; 1 cycle at 4 °C for 5 min, and 1 cycle at 90 °C for 5 min all at a ramp rate of 2 °C/s on a Bio-Rad T100 thermal cycler was used for the PCR step. Droplets quantified using the Bio-Rad Quantisoft software. A total of two replicates were used per sample.

### 450k Array methylome clustering and copy-number profiling

DNA methylation was analyzed by the Illumina Infinium HumanMethylation450 (450k) array assessing 482,421 CpG sites (Illumina, San Diego, USA), according to the manufacturer’s instructions at the NYU molecular laboratory. DNA methylation data were normalized by performing background correction and dye bias correction (shifting of negative control probe mean intensity to zero and scaling of normalization control probe mean intensity to 10,000, respectively). Filtering of probes was performed as described previously^4,28^ (removal of probes targeting sex chromosomes, containing single nucleotide polymorphism and not uniquely matched). To enable future comparability, we also removed probes not represented on the novel Illumina Infinium HumanMethylation EPIC array. In total, 428,799 probes were kept for analysis. For unsupervised hierarchical clustering, we selected 5000 probes that showed the highest standard deviation across the beta values. Samples were hierarchically clustered using 1-Pearson correlation as a distance measure and average linkage as agglomeration method. The unscaled methylation levels were shown in a heat map from unmethylated state (blue color) to methylated state (red color). Copy-number profiles were generated using the “conumee” R package in Bioconductor (http://www.bioconductor.org/packages/release/bioc/html/conumee.html), and assessed manually.

### Whole-transcriptome sequencing and analysis

RNA was extracted from FFPE tissue using the automated Maxwell system (Promega, Madison, USA). RNASeq libraries were prepared using the Illumina TruSeq Stranded Total RNA library prep, with Ribozero Gold, starting from 500 ng of DNAse I treated total RNA, following the manufacturer’s protocol, with the exception that 14 cycles of PCR were performed to amplify the libraries, to keep the duplication rate lower than with the recommended 15 cycles. The amplified libraries were purified using AMPure beads, quantified by Qubit and QPCR, and visualized in an Agilent Bioanalyzer. The libraries were pooled equimolarly, and loaded at 8 pm, on a high output HiSeq 2500 flow cell, as paired 50 nucleotide reads. Sequencing results were demultiplexed and converted to FASTQ format using Illumina bcl2fastq software. The sequencing reads were aligned to the human genomes (build hg19/GRCh37) using the splice-aware STAR aligner^29^. The featureCounts program^30^ was utilized to generate counts for each gene based on how many aligned reads overlap its exons. These counts were then normalized and used to test for differential expression using negative binomial generalized linear models implemented by the DESeq2 R package^31^.

### miRNA analysis

Extracted RNA quality and quantity were analyzed on an agilent Bioanalyzer 2100 using a nano chip. miRNA was hybridized using the Nanostring nCounter® Human v3A miRNA Expression Assay, according to the manufacturer’s protocol. A total of 100 ng RNA were used per sample. Hybridizations were processed on the nCounter Prep Station, and prepped cartridges were scanned on the Nanostring Digital Analyzer using 280 field of view counts. Data were analyzed with Nanostring nSolver 3.0. Samples were analyzed using the default quality control measures, and normalized to ligation controls in the Nanostring codesets.

### Immunohistochemistry

Unconjugated polyclonal rabbit anti-human DUF1220 (Millipore Cat# AB15369 Lot# 2479814 RRID:AB_11214420) generated against a synthetic peptide from primates; and unconjugated polyclonal rabbit anti-human cardiomyopathy-associated protein 2 (PDE4DIP, Abcam Cat# ab121375 Lot# GR191210-1 RRID:AB_11128871) raised against a recombinant fragment, corresponding to amino acids 26–171 of human PDE4DIP isoform 2, were used for immunohistochemistry. Paraffin-embedded tissues were sectioned at 4-µm. Chromogenic immunohistochemistry was performed on a Ventana Medical Systems Discovery XT instrument with online deparaffinization using Ventana’s reagents and detection kits unless otherwise noted. PDE4DIP was antigen retrieved in Ventana Cell Conditioner 1 (Tris-Borate-EDTA), and DUF1220 was antigen retrieved in Ventana Cell Conditioner 2 (Citrate) for 20 min, respectively. Both antibodies were diluted 1:100 in Dulbecco’s phosphate buffered saline (Invitrogen/Life Technologies), and incubated for 3 h at room temperature. Primary antibody was detected with anti-rabbit, horseradish peroxidase conjugated multimer incubated for 8 min. The complex was visualized with 3,3 diaminobenzidene and enhanced with copper sulfate. Slides were washed in distilled water, counterstained with hematoxylin, dehydrated, and mounted with permanent media. Negative controls were incubated with Dulbecco’s phosphate buffered saline instead of primary antibody and did not show any signal. Immunohistochemistry was performed on 12 PB samples for which FFPE material was available and 15 normal control samples, 5 from fetal autopsy and 5 from adult fetopsy brains and their respective pineal glands. Tissue from fetal pineal glands were not available for analysis.

### Generation and validation of DROSHA knock-down in hNSC

Targeting *DROSHA* with LentiCRISPR: Genetic disruption of *DROSHA* was achieved by cloning small guide RNAs (sgRNAs), targeting DROSHA into lentiCRISPR-V2 vector (Addgene Plasmid #52961). Briefly, oligonucleotides for sgRNAs were synthesized by Integrated DNA Technologies, annealed in vitro and ligated into a Bsmb1 digested lentiCRISPR-V2 vector (sgRNA: Sense-CACC G TACAGATCTGATTATGACCG, antisense-AAAC CGGTCATAATCAGATCTGTAC). The product was then used to generate lentivirus-containing supernatant that was concentrated by ultracentrifugation at 25000xg for 2 h at 4 C (40 mL total). Concentrated virus was resuspended in 50 µL of PBS.

Cell culture, transduction, and selection: hNSC were generated and maintained as in Pourchet et al^32^. These hNSCs have been characterized by RNA-seq, 450 K array DNA methylation profiling, and karyotype^33^. They are functionally multipotent, and can be directed to differentiate to neuroglial lineages. Briefly, hNSC were cultured in media supplemented with EGF and FGF2 and maintained on poly-ornithine/laminin coated plates. Cultures were routinely tested for mycoplasma contamination. One well in a six-well plate of hNSC were transduced with 2–8 µL of packaged lentiCRISPR-V2 per well for 10 h, at which time cells were washed and media changed every 48 h. Seventy-two hours later, cells were cultured in 0.3 µg/mL of puromycin for 7 days to generate only puromycin-resistant colonies. After selection, cells were passaged in 0.1 µg/mL of puromycin and then maintained in the absence of puromycin. This lentiCRISPR targeting of DROSHA was performed in triplicate.

Western blot: Successful Lenti-Cas9 expression and DROSHA knockdown were confirmed by Western blot. Protein lysate was prepared from Wild type (WT) and the triplicate DROSHA LentiCRISPR transduced hNSC lines using an 8 M urea, 1% CHAPS buffer. Twenty micrograms of protein lysate was run per lane and resolved on a 4–12% Bis-Tris gel. After transfer, membranes were probed using antibodies directed at Cas9 (1:1000; Millipore MAC133), DROSHA (1:1000; Abcam 183732), GAPDH (1:5000; GeneTex 627408), H3 (1:5000; Abcam 1791). Visualization was performed using HRP-conjugated secondary antibodies directed against the appropriate primary antibody host, as developed using chemiluminescence. Average band intensity in each lane was measured for DROSHA, GAPDH, and histone H3 using ImageJ software. DROSHA intensity was normalized to the average of the GAPDH and H3 control band intensities.

Sanger sequencing: Primers were designed to amplify a 217 bp fragment of the *DROSHA* locus that encompassed the gRNA target in Exon 4 (FWD Primer: CTATGATGACCACAGGCACCGA, REV Primer: GGCCTCTGCAGTTCATTAAAG). Following standard qPCR amplification of this product, Sanger sequencing was performed for each of the three LentiCRISP hNSC lines generated.

### Data availability

All original sequencing reads have been deposited in NCBI’s public repository Sequencing Reads Archive (SRA). They are available under the BioProject ID: PRJNA446027.

In particular, the bam files are available under the accession ids SAMN08805007, SAMN08805008, SAMN08805009, SAMN08805010, SAMN08805011, SAMN08805012, SAMN08805013, SAMN08805014, SAMN08805015, SAMN08805016, SAMN08805017, SAMN08805018, SAMN08805019, SAMN08805020, SAMN08805021, SAMN08805022, SAMN08805023, SAMN08805024, SAMN08805025, and SAMN08805026.

No restrictions have been placed on data availability. Data should be available immediately after processing (by SRA).

## Electronic supplementary material

Supplementary Information
