# Supplementary material for: Recurrent homozygous deletion of DROSHA and microduplication of PDE4DIP in pineoblastoma
Source: Nat Commun. 2018 Jul 20;9:2868. doi: 10.1038/s41467-018-05029-3 (PMC6054684; doi:10.1038/s41467-018-05029-3)
Supplement: Supplementary file 1 — Supplementary Information [file 41467_2018_5029_MOESM1_ESM.pdf]

Supplementary Figures and Tables

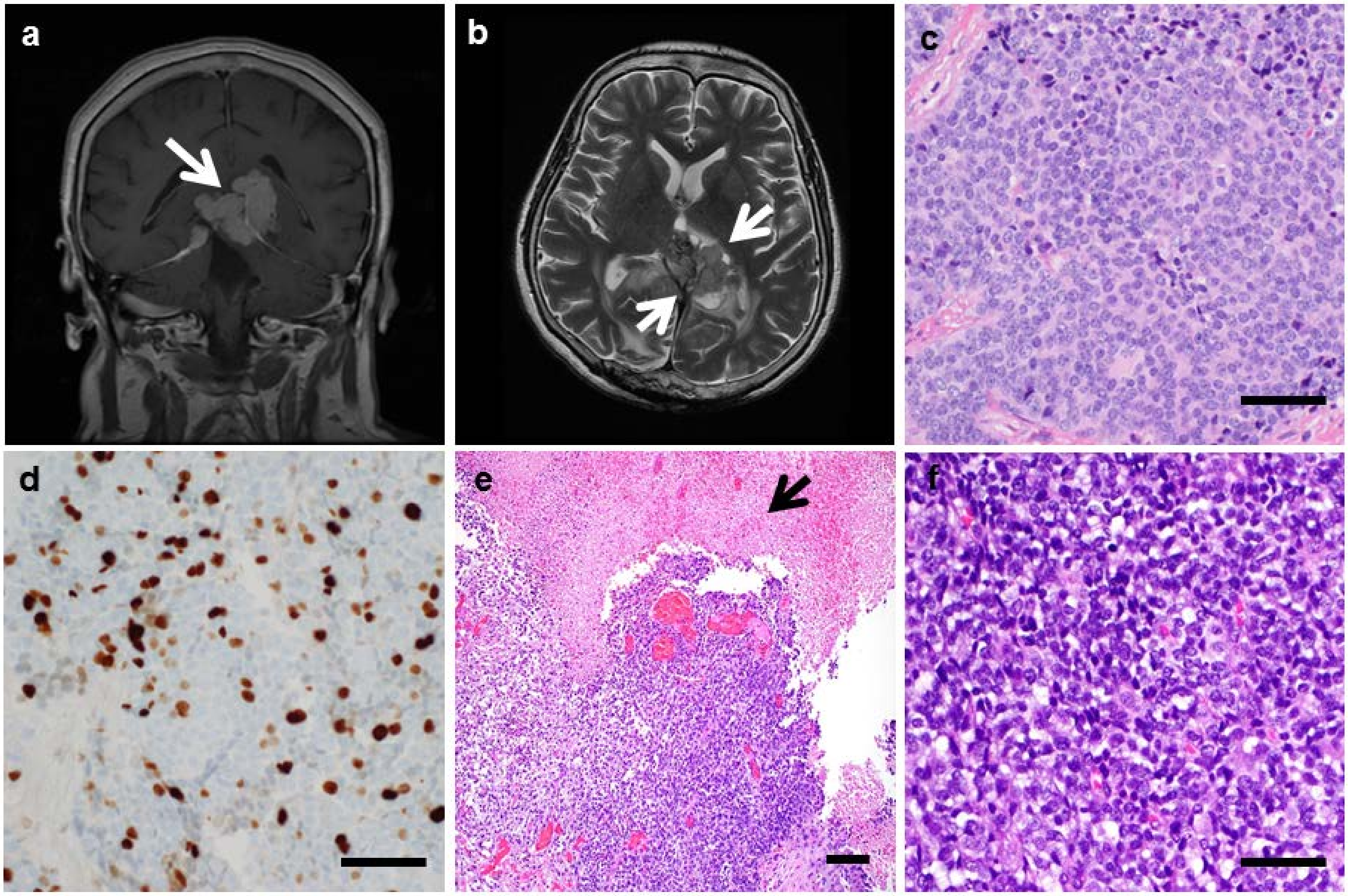

Supplementary Figure 1: Clinical and pathological features of adult pineoblastoma. Pineoblastomas of adults are indistinguishable from pediatric pineoblastoma by stadard diagnostic methods. A 69-year-old woman presented with rapidly progressive visual loss resulting in blindness in both eyes. Her MRI scan at that time showed marked hydrocephalus and a pineal region mass, which was biopsied and diagnosed as pineoblastoma. (a,b) Preoperative MRI: coronal T1 post-contrast (a), axial T2 (b). Arrows indicate the tumor. (c): Hematoxylin and Eosin (H&E) section of the tumor at the time of diagnosis showed a classic pattern of primitive neuroectodermal tumor with sheets of small round blue cells. (d): Tumor showed brisk mitotic activity and high proliferation index highlighted by Ki-67. The patient underwent radiation therapy receiving 6000 cGy but progressed within 1 year and underwent second surgery with subtotal resection again showing pineoblastoma with areas of necrosis (e, arrow) and largely viable diffuse sheets of small round blue cells similar to the original tumor (f). The patient rapidly deteriorated and was referred to hospice care. Scale bar 100 µm.

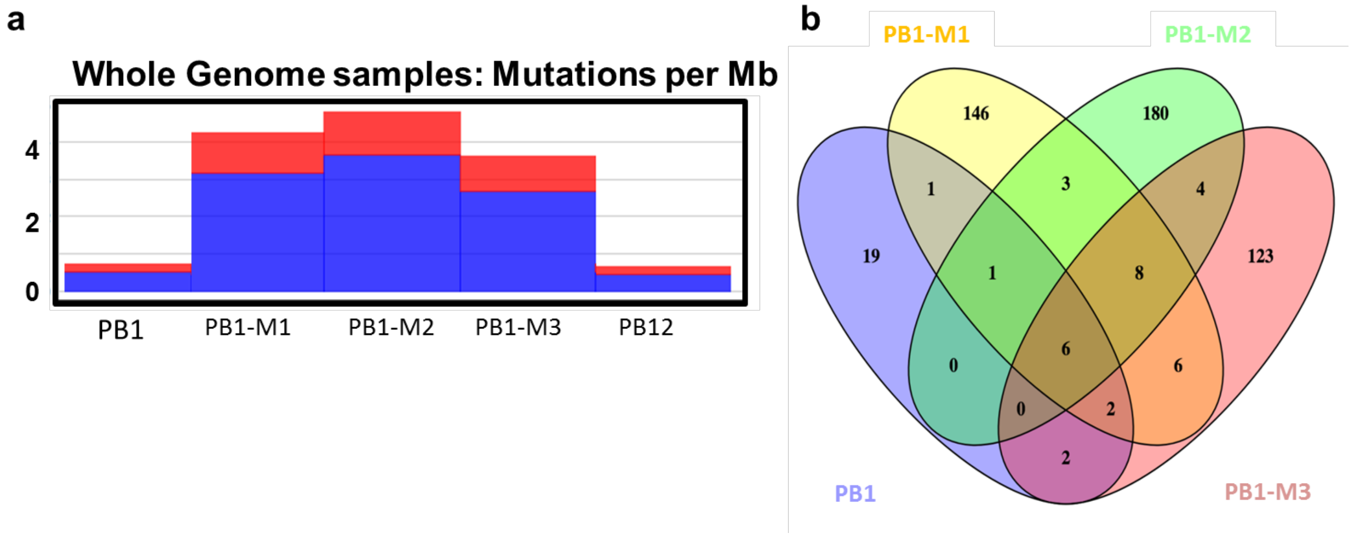

Supplementary Figure 2: Mutational burden in pineoblastoma: (a) WGS (matched Tumor and Germline). Somatic mutation rate in pineoblastomas (PB1 and PB12) is relatively low, similar to other pediatric cancers. (b) Venn diagram showing a comparison of somatic mutations shared among PB1 at the time of diagnosis and metastatic lesions collected at the time of autopsy shows markedly increase number of mutations in metastatic lesions (a and b). However, the patient underwent several cycles of chemotherapy and radiation, which may have contributed to increased mutation rate. Red: synonymous mutations, Blue: non-synonymous mutations

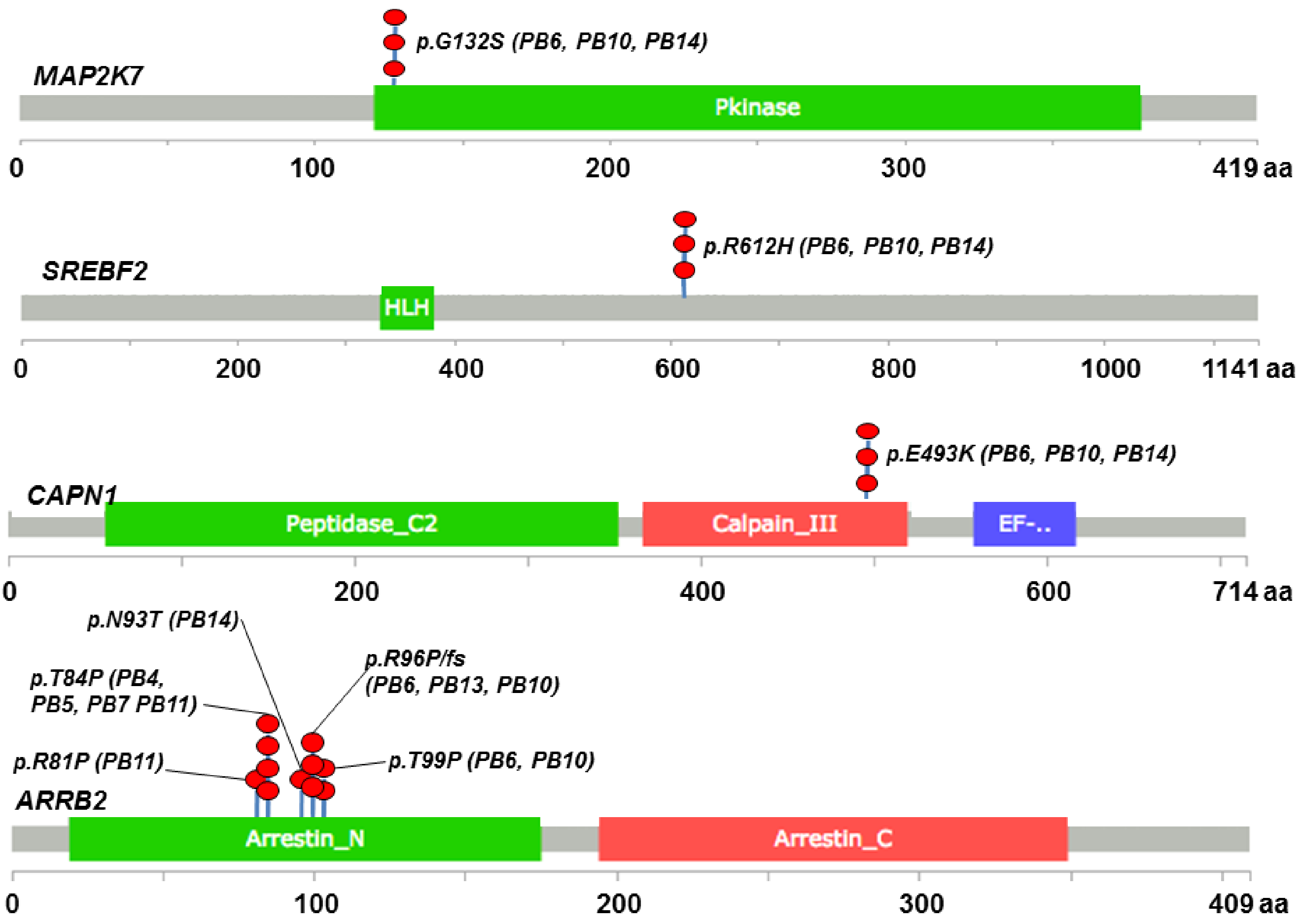

Supplementary Figure 3: Variants identified by WES in tumor only samples of pineoblastomas: *MAP2K7*, *SREBF2*, *CAPN1* and *ARRB2* were most commonly mutated genes in WES samples identified by pathway enrichment analysis. However, the lack of germline in these tumors precludes defining these variants as somatic mutations.

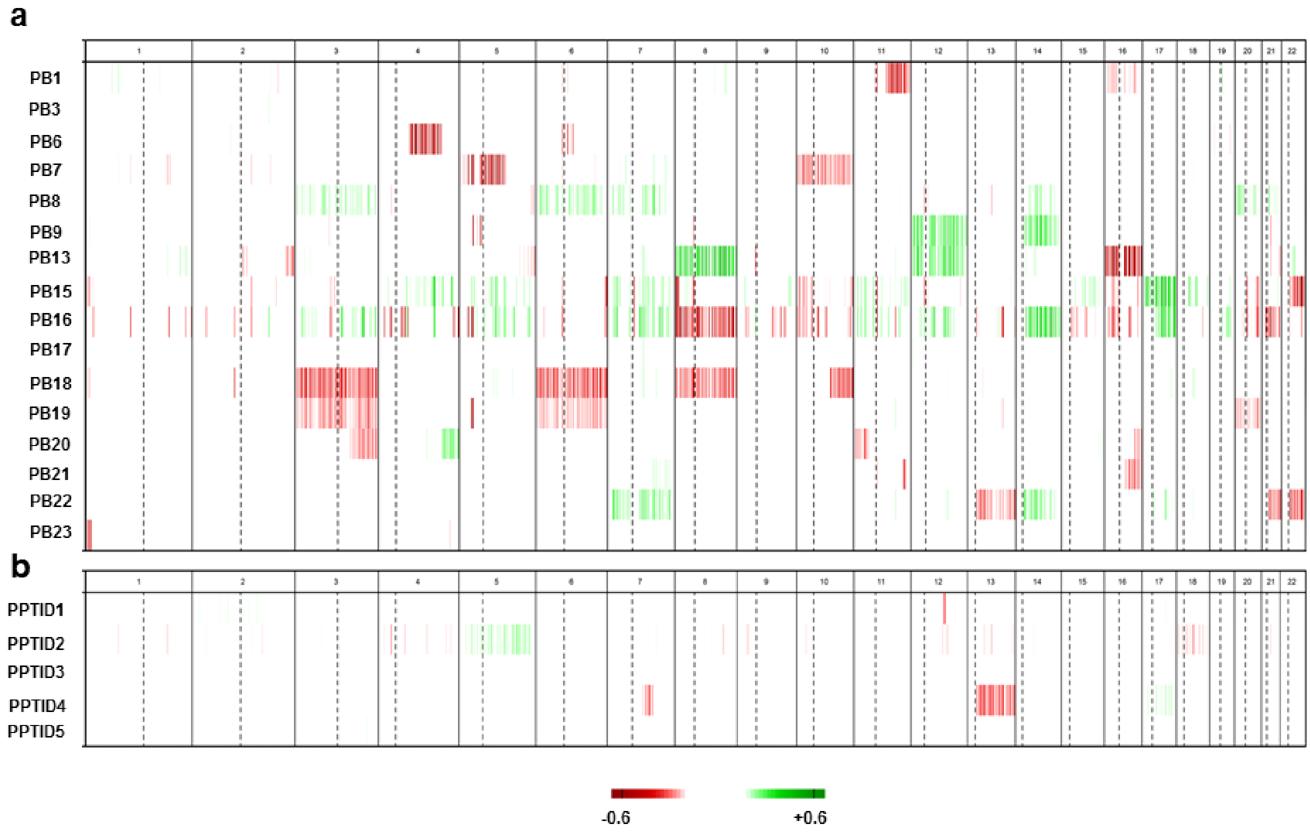

Supplementary Figure 4: Copy number landscape of pineoblastoma (PB) and pineal parenchymal tumor of intermediate differentiation (PPTID). Pineoblastomas showed random numerical abnormalities involving numerous chromosomes. In contrast, tumors that clustered with PPTID and adult PB (3, 8, and 23) showed fewer numerical aberrations.

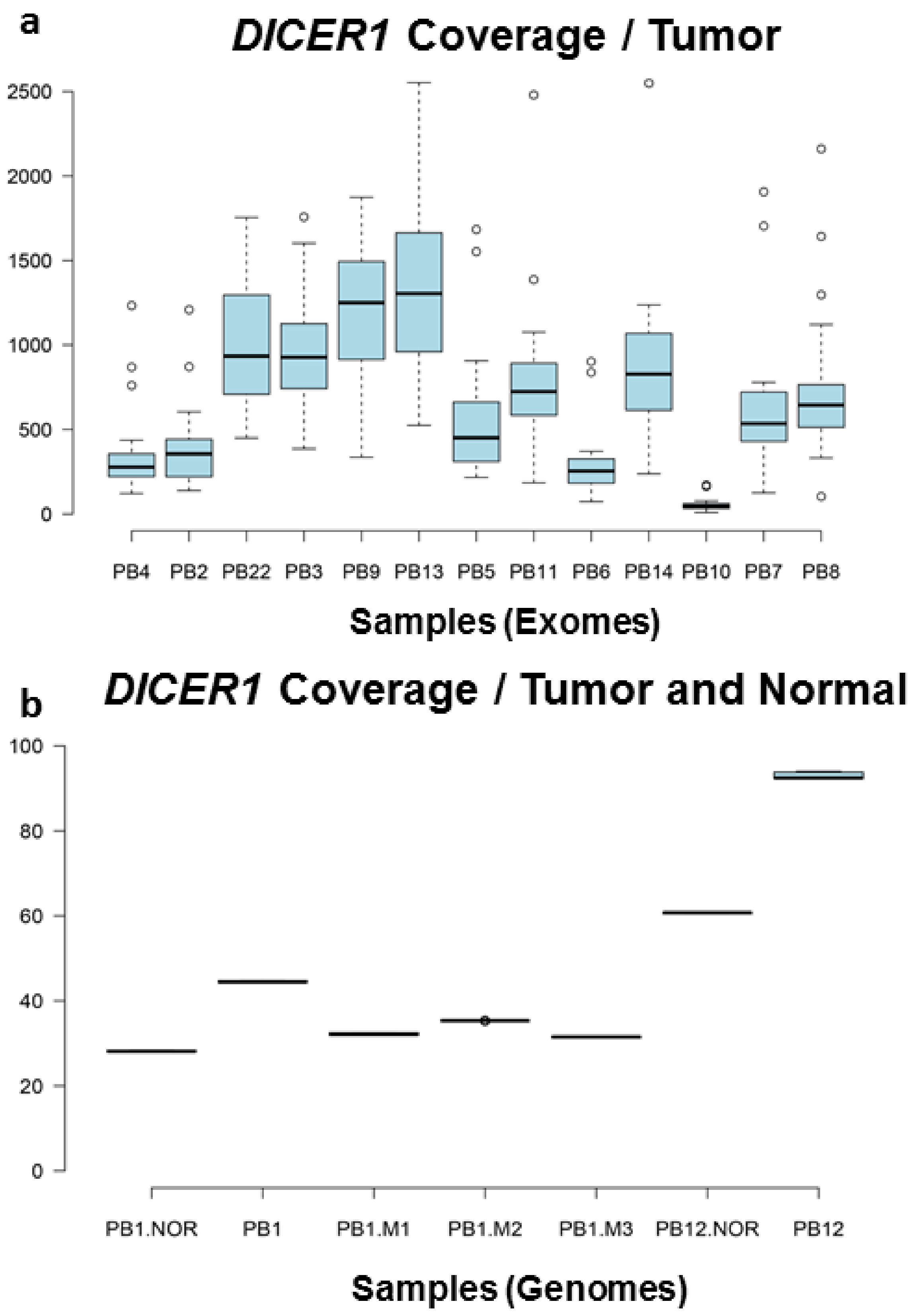

Supplementary Figure 5: Genomic sequencingcoverage of *DICER1* in (a) WES samples and (b) WGS samples (NOR: matched normal germline). No mutations were identified in the *DICER1*.

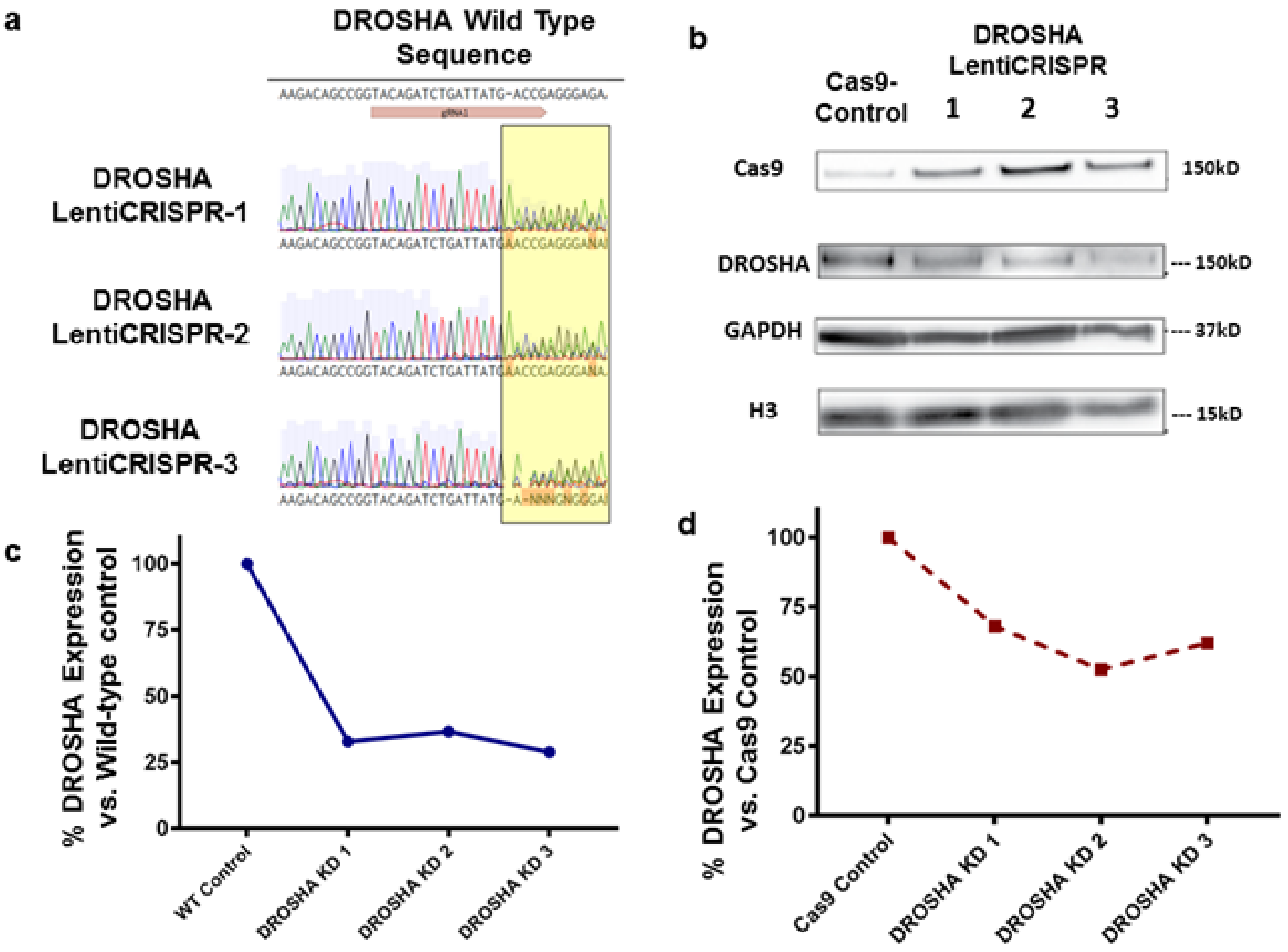

Supplementary Figure 6: CRISPR-targeting of *DROSHA* in hNSC lines: (a) Genetic confirmation of the CRISPR-mediated disruption of Exon 4 of the *DROSHA* locus: The wild-type sequence is shown at the top with the location of gRNA target below. Sanger sequencing is aligned to the wild-type template for each of the cell lines using Benchling Software. All *DROSHA* LentiCRISPR show mixed spectra beginning at a consistent location within the gRNA indicative of frameshift insertion/deletions at the locus disrupting the protein coding of DROSHA within the population. This corresponds to observed decrease of the DROSHA protein by western blot in (b) and Figure 5c. (c-d) Average band intensity in each lane was measured for DROSHA, GAPDH and Histone H3 using ImageJ Software. DROSHA intensity was normalized to the average of the GAPDH and H3 control band intensities. (c) The % DROSHA expression vs. wild-type (see Fig. 5c) was calculated for each of the LentiCRISPR cell lines revealing a robust reduction of ~70% of the DROSHA protein levels across cell lines.(d) In a separate experiment, a control hNSC cell line was generated containing the LentiCRISPR vector with a gRNA sequence that targets the Tomato locus (Cas9-Control) showing reduction of DROSHA protein, confirming that the decrease of DROSHA does not represent a nonspecific effect of the lentivirus infection.

Gene	CDNA	AA Change	Impact	Sample	Exome/ Genome	Chr	Position
ABCA2	c.C5636T	p.T1879I	nonsynonymous SNV	PB1-M2	Genome	chr9	139906196
ABCD2	c.C2084A	p.T695K	nonsynonymous SNV	PB1-M2	Genome	chr12	39947853
ACOT6	c.G291T	p.L97F	nonsynonymous SNV	PB1-M1	Genome	chr14	74086210
ACOT6	c.G291T	p.L97F	nonsynonymous SNV	PB1-M2	Genome	chr14	74086210
ACOT6	c.G291T	p.L97F	nonsynonymous SNV	PB1-M3	Genome	chr14	74086210
AGO3	c.A1012C	p.T338P	nonsynonymous SNV	PB8	Exome	chr1	36474628
ALPK2	c.T2686G	p.L896V	nonsynonymous SNV	PB1-M2	Genome	chr18	56204733
ARID3A	c.418_420del	p.140_140del	inframe deletion	PB10	Exome	chr19	932467
ARID3A	c.C657A	p.D219E	nonsynonymous SNV	PB5	Exome	chr19	932706
ARRB2	c.289dupC	p.R96fs	frameshift insertion	PB10	Exome	chr17	4619834
ARRB2	c.A250C	p.T84P	nonsynonymous SNV	PB11	Exome	chr17	4619841
ARRB2	c.A250C	p.T84P	nonsynonymous SNV	PB4	Exome	chr17	4619841
ARRB2	c.A250C	p.T84P	nonsynonymous SNV	PB5	Exome	chr17	4619841
ARRB2	c.A250C	p.T84P	nonsynonymous SNV	PB7	Exome	chr17	4619841
ARRB2	c.A278C	p.N93T	nonsynonymous SNV	PB14	Exome	chr17	4619824
ARRB2	c.A295C	p.T99P	nonsynonymous SNV	PB10	Exome	chr17	4619841
ARRB2	c.A295C	p.T99P	nonsynonymous SNV	PB6	Exome	chr17	4619841
ARRB2	c.G242C	p.R81P	nonsynonymous SNV	PB11	Exome	chr17	4619833
ARRB2	c.G287C	p.R96P	nonsynonymous SNV	PB13	Exome	chr17	4619833

ARRB2	c.G287C	p.R96P	nonsynonymous SNV	PB6	Exome	chr17	4619833
ASTL	c.G1130A	p.G377D	nonsynonymous SNV	PB1-M2	Genome	chr2	96789755
BARD1	c.C1304T	p.A435V	nonsynonymous SNV	PB4	Exome	chr2	215645294
BDP1	c.A5163T	p.K1721N	nonsynonymous SNV	PB1-M2	Genome	chr5	70818287
BRF1	c.A496T	p.M166L	nonsynonymous SNV	PB3	Exome	chr14	105693045
BTBD6	c.C911T	p.T304I	nonsynonymous SNV	PB7	Exome	chr14	105716462
C12orf51	c.G12471T	p.E4157D	nonsynonymous SNV	PB1-M1	Genome	chr12	112601370
C12orf51	c.C12785G	p.S4262C	nonsynonymous SNV	PB1-M3	Genome	chr12	112600261
C5orf42	c.T8925A	p.S2975S	synonymous SNV	PB1-M1	Genome	chr5	37121817
C5orf42	c.A8669G	p.D2890G	nonsynonymous SNV	PB1-M2	Genome	chr5	37125473
CAPN1	c.G1477A	p.E493K	nonsynonymous SNV	PB10	Exome	chr11	64974057
CAPN1	c.G1477A	p.E493K	nonsynonymous SNV	PB14	Exome	chr11	64974057
CAPN1	c.G1477A	p.E493K	nonsynonymous SNV	PB6	Exome	chr11	64974057
CHD8	c.C3740T	p.S1247F	nonsynonymous SNV	PB4	Exome	chr14	21868460
CHD8	c.6275delA	p.N2092fs	frameshift deletion	PB5	Exome	chr14	21859176
CHD8	c.C3820T	p.R1274W	nonsynonymous SNV	PB8	Exome	chr14	21868380
COL2A1	c.C3134T	p.P1045L	nonsynonymous SNV	PB1	Genome	chr12	48371414
COL2A1	c.C3134T	p.P1045L	nonsynonymous SNV	PB1-M1	Genome	chr12	48371414
COL2A1	c.C3134T	p.P1045L	nonsynonymous SNV	PB1-M2	Genome	chr12	48371414
COL2A1	c.C3134T	p.P1045L	nonsynonymous SNV	PB1-M3	Genome	chr12	48371414

CREBBP	c.6645_6647del	p.2215_2216del	inframe deletion	PB5	Exome	chr16	3778401
CREBBP	c.C6509T	p.A2170V	nonsynonymous SNV	PB7	Exome	chr16	3778539
CREBBP	c.G2225C	p.R742P	nonsynonymous SNV	PB8	Exome	chr16	3824628
CREG1	c.C83T	p.P28L	nonsynonymous SNV	PB3	Exome	chr1	167522896
CSMD2	c.A5476G	p.I1826V	nonsynonymous SNV	PB1-M3	Genome	chr1	34090148
CYP4F2	c.C1448G	p.A483G	nonsynonymous SNV	PB1-M2	Genome	chr19	15989696
CYSLTR1	c.G25T	p.V9L	nonsynonymous SNV	PB12	Genome	chrX	77529219
DCHS2	c.T6734A	p.I2245N	nonsynonymous SNV	PB1-M1	Genome	chr4	155157705
DCHS2	c.T6734A	p.I2245N	nonsynonymous SNV	PB1-M2	Genome	chr4	155157705
DCHS2	c.T6734A	p.I2245N	nonsynonymous SNV	PB1-M3	Genome	chr4	155157705
DDX17	c.A1511G	p.H504R	nonsynonymous SNV	PB3	Exome	chr22	38884057
DNAH6	c.A11467C	p.N3823H	nonsynonymous SNV	PB1-M2	Genome	chr2	85024768
ECD	c.C1094A	p.P365H	nonsynonymous SNV	PB5	Exome	chr10	74899400
EEF1A1	c.G877A	p.E293K	nonsynonymous SNV	PB13	Exome	chr6	74228229
EEF1A1	c.G943C	p.V315L	nonsynonymous SNV	PB5	Exome	chr6	74228163
ELN	c.G2000C	p.C667S	nonsynonymous SNV	PB1-M1	Genome	chr7	73482996
ELN	c.G2141C	p.C714S	nonsynonymous SNV	PB1-M3	Genome	chr7	73482996
ENPP2	c.A1922T	p.E641V	nonsynonymous SNV	PB1-M2	Genome	chr8	120581606
ERBB2IP	c.G2835C	p.K945N	nonsynonymous SNV	PB2	Exome	chr5	65349981

FBN1	c.G4568A	p.R1523Q	nonsynonymous SNV	PB1	Genome	chr15	48760623
FCER1G	c.G152A	p.R51Q	nonsynonymous SNV	PB7	Exome	chr1	161188041
FGFR1	c.C581T	p.P194L	nonsynonymous SNV	PB4	Exome	chr8	38282109
FOS	c.C510A	p.D170E	nonsynonymous SNV	PB5	Exome	chr14	75747494
GATA1	c.C1168T	p.P390S	nonsynonymous SNV	PB7	Exome	chrX	48652497
GHR	c.T1196C	p.F399S	nonsynonymous SNV	PB5	Exome	chr5	42718805
GPAT2	c.C2074T	p.R692C	nonsynonymous SNV	PB1	Genome	chr2	96688929
GPX5	c.T299A	p.M100K	nonsynonymous SNV	PB1	Genome	chr6	28500155
GPX5	c.T299A	p.M100K	nonsynonymous SNV	PB1-M1	Genome	chr6	28500155
GPX5	c.T299A	p.M100K	nonsynonymous SNV	PB1-M2	Genome	chr6	28500155
GPX5	c.T299A	p.M100K	nonsynonymous SNV	PB1-M3	Genome	chr6	28500155
HBS1L	c.G504C	p.E168D	nonsynonymous SNV	PB1-M3	Genome	chr6	135318704
HINT1	c.C151T	p.H51Y	nonsynonymous SNV	PB4	Exome	chr5	130498330
HLA-DRA	c.A119T	p.N40I	nonsynonymous SNV	PB1-M2	Genome	chr6	32410261
JARID2	c.T1235A	p.F412Y	nonsynonymous SNV	PB2	Exome	chr6	15497207
JARID2	c.G2164A	p.V722I	nonsynonymous SNV	PB9	Exome	chr6	15507596
KAT6A	c.G4828A	p.A1610T	nonsynonymous SNV	PB10	Exome	chr8	41790910
KAT6A	c.T1195C	p.S399P	nonsynonymous SNV	PB2	Exome	chr8	41834694
KAT6A	c.T1193C	p.V398A	nonsynonymous SNV	PB2	Exome	chr8	41834696

KCNA5	c.C355A	p.H119N	nonsynonymous SNV	PB1-M2	Genome	chr12	5153668
KCNN4	c.C1084T	p.R362W	nonsynonymous SNV	PB12	Genome	chr19	44273150
KRTAP4-4	c.T202C	p.S68P	nonsynonymous SNV	PB1-M2	Genome	chr17	39316742
LIG1	c.G2143T	p.V715L	nonsynonymous SNV	PB1	Genome	chr19	48626437
LY75-CD302	c.A2411G	p.Y804C	nonsynonymous SNV	PB1-M2	Genome	chr2	160711423
MAP2K7	c.G394A	p.G132S	nonsynonymous SNV	PB10	Exome	chr19	7975205
MAP2K7	c.G394A	p.G132S	nonsynonymous SNV	PB14	Exome	chr19	7975205
MAP2K7	c.G394A	p.G132S	nonsynonymous SNV	PB6	Exome	chr19	7975205
MGLL	c.C187T	p.R63W	nonsynonymous SNV	PB1	Genome	chr3	127500677
MGLL	c.C187T	p.R63W	nonsynonymous SNV	PB1-M1	Genome	chr3	127500677
MGLL	c.C187T	p.R63W	nonsynonymous SNV	PB1-M2	Genome	chr3	127500677
MGLL	c.C187T	p.R63W	nonsynonymous SNV	PB1-M3	Genome	chr3	127500677
MUC13	c.G1177A	p.G393S	nonsynonymous SNV	PB13	Exome	chr3	124631992
MYH3	c.A1529T	p.D510V	nonsynonymous SNV	PB1-M1	Genome	chr17	10546195
MYH3	c.A1529T	p.D510V	nonsynonymous SNV	PB1-M3	Genome	chr17	10546195
MYO3A	c.T2975C	p.I992T	nonsynonymous SNV	PB1-M2	Genome	chr10	26446420
NBPF14	c.G319A	p.V107I	nonsynonymous SNV	PB1-M1	Genome	chr1	148023637
NFAT5	c.T1250C	p.I417T	nonsynonymous SNV	PB3	Exome	chr16	69693683
NFKB2	c.C1787A	p.P596H	nonsynonymous SNV	PB3	Exome	chr10	104160237

NFKB2	c.C1786T	p.P596S	nonsynonymous SNV	PB3	Exome	chr10	104160236
NFKB2	c.C760A	p.Q254K	nonsynonymous SNV	PB4	Exome	chr10	104157836
OR1D5	c.T353C	p.M118T	nonsynonymous SNV	PB1	Genome	chr17	2966549
OR52H1	c.G586T	p.A196S	nonsynonymous SNV	PB1	Genome	chr11	5566168
POU1F1	c.T686A	p.L229Q	nonsynonymous SNV	PB1-M2	Genome	chr3	87309234
POU6F1	c.C335G	p.S112C	nonsynonymous SNV	PB1-M2	Genome	chr12	51586169
PPP1CC	c.A725C	p.D242A	nonsynonymous SNV	PB2	Exome	chr12	111160299
PRAMEF11	c.G683A	p.R228H	nonsynonymous SNV	PB1-M2	Genome	chr1	12887174
PRKCD	c.G1444A	p.D482N	nonsynonymous SNV	PB4	Exome	chr3	53222764
PSMC4	c.A242C	p.Y81S	nonsynonymous SNV	PB11	Exome	chr19	40480213
PTEN	c.A45T	p.R15S	nonsynonymous SNV	PB2	Exome	chr10	89624271
PTK2B	c.T1316C	p.V439A	nonsynonymous SNV	PB1	Genome	chr8	27293840
PTK2B	c.T1316C	p.V439A	nonsynonymous SNV	PB1-M3	Genome	chr8	27293840
RASA3	c.C1658T	p.A553V	nonsynonymous SNV	PB1	Genome	chr13	114774873
RASSF1	c.T515C	p.L172P	nonsynonymous SNV	PB3	Exome	chr3	50369247
RBBP6	c.C2275T	p.R759C	nonsynonymous SNV	PB2	Exome	chr16	24580388
RDBP	c.G661A	p.D221N	nonsynonymous SNV	PB1-M1	Genome	chr6	31922413
RDBP	c.A662T	p.D221V	nonsynonymous SNV	PB1-M1	Genome	chr6	31922412
RDBP	c.G661A	p.D221N	nonsynonymous SNV	PB1-M2	Genome	chr6	31922413

RDBP	c.A662T	p.D221V	nonsynonymous SNV	PB1-M2	Genome	chr6	31922412
RDH16	c.C225A	p.D75E	nonsynonymous SNV	PB1-M2	Genome	chr12	57351022
SALL3	c.G1717A	p.V573M	nonsynonymous SNV	PB1	Genome	chr18	76753708
SERPINB5	c.C1095A	p.N365K	nonsynonymous SNV	PB2	Exome	chr18	61170922
SETD8	c.C85A	p.P29T	nonsynonymous SNV	PB5	Exome	chr12	123874054
SLC38A10	c.A1931G	p.E644G	nonsynonymous SNV	PB1-M2	Genome	chr17	79226009
SP110	c.T2034A	p.H678Q	nonsynonymous SNV	PB11	Exome	chr2	231033876
SREBF2	c.G1835A	p.R612H	nonsynonymous SNV	PB10	Exome	chr22	42276793
SREBF2	c.G1835A	p.R612H	nonsynonymous SNV	PB14	Exome	chr22	42276793
SREBF2	c.G1835A	p.R612H	nonsynonymous SNV	PB6	Exome	chr22	42276793
TCF4	c.C460G	p.P154A	nonsynonymous SNV	PB2	Exome	chr18	53018138
TCF7L2	c.C1549T	p.L517F	nonsynonymous SNV	PB2	Exome	chr10	114925540
TET1	c.T4973G	p.F1658C	nonsynonymous SNV	PB1-M1	Genome	chr10	70442651
TET1	c.T4973G	p.F1658C	nonsynonymous SNV	PB1-M2	Genome	chr10	70442651
TET1	c.T4973G	p.F1658C	nonsynonymous SNV	PB1-M3	Genome	chr10	70442651
TGFBR1	c.G974T	p.G325V	nonsynonymous SNV	PB5	Exome	chr9	101907014
TOP2B	c.G79C	p.A27P	nonsynonymous SNV	PB1-M2	Genome	chr3	25686937
TRH	c.T373C	p.W125R	nonsynonymous SNV	PB9	Exome	chr3	129695703
TSHZ3	c.G1288A	p.V430I	nonsynonymous SNV	PB12	Genome	chr19	31769411

TTN	c.T43647G	p.S14549R	nonsynonymous SNV	PB1-M2	Genome	chr2	179440017
TXLNA	c.C821A	p.T274N	nonsynonymous SNV	PB1-M2	Genome	chr1	32655708
VTN	c.C634T	p.R212C	nonsynonymous SNV	PB5	Exome	chr17	26696345
XRCC6	c.T117G	p.I39M	nonsynonymous SNV	PB9	Exome	chr22	42024156
ZDHHC11	c.C908A	p.A303D	nonsynonymous SNV	PB1-M1	Genome	chr5	833915
ZNF292	c.G7202A	p.S2401N	nonsynonymous SNV	PB1-M3	Genome	chr6	87970549
ZNF583	c.T268C	p.S90P	nonsynonymous SNV	PB1-M2	Genome	chr19	56934295
ZNF789	c.C1186A	p.P396T	nonsynonymous SNV	PB1-M1	Genome	chr7	99085019
ZNF789	c.C1186A	p.P396T	nonsynonymous SNV	PB1-M2	Genome	chr7	99085019
ZNF789	c.C1186A	p.P396T	nonsynonymous SNV	PB1-M3	Genome	chr7	99085019
ZNF8	c.T443A	p.L148Q	nonsynonymous SNV	PB1-M2	Genome	chr19	58805617

Supplementary Table 1. Mutations identified by WES and WGS in pineoblastoma samples.

gene	baseMean	log2FC	pvalue	padj
CACNA1E	7812.5	7.70	0.0000000000	0.0000000000
CTCFL	3547.3	-8.07	0.0000000000	0.0000000000
TXLNG2P	1933.9	-7.96	0.0000000000	0.0000000000
TMEM132D	796	7.58	0.0000000000	0.0000000000
RPS4Y1	3286.4	-7.12	0.0000000000	0.0000000000
SKOR1	3809.3	6.95	0.0000000000	0.0000000000
CTD-2281M20.1	2129.2	-7.08	0.0000000000	0.0000000000
BHLHE41	717.7	7.61	0.0000000000	0.0000000000
TMEM26	505	7.49	0.0000000000	0.0000000000
IGF2BP1	1683.9	-7.64	0.0000000000	0.0000000000
GPR155	1312.3	6.58	0.0000000000	0.0000000000
CA10	2895.6	-6.59	0.0000000000	0.0000000000
USP9Y	1455.8	-6.58	0.0000000000	0.0000000000
KDM5D	1366.7	-6.60	0.0000000000	0.0000000000
MAGEC1	848.3	-8.32	0.0000000000	0.0000000000
DDX3Y	1995.2	-6.29	0.0000000000	0.0000000000
PPEF1	1716	-6.71	0.0000000000	0.0000000000
UTY	882.1	-6.97	0.0000000000	0.0000000000
NNAT	816.5	-7.70	0.0000000000	0.0000000000
ATOH8	441.9	6.41	0.0000000000	0.0000000000
RBM46	950.3	-6.49	0.0000000000	0.0000000000
LRRC7	1256.1	-6.33	0.0000000000	0.0000000000
RP11-343H5.4	1092.6	-6.83	0.0000000000	0.0000000000
TTTY15	566.6	-6.95	0.0000000000	0.0000000000
PTCSC3	175.7	7.76	0.0000000000	0.0000000000
ADM	1054.9	5.74	0.0000000000	0.0000000000

CA12	1612.5	6.85	0.0000000000	0.0000000000
TPTEP1	385.8	6.10	0.0000000000	0.0000000000
FOXA2	154.3	8.13	0.0000000000	0.0000000000
NAP1L5	266.4	6.57	0.0000000000	0.0000000000
GALR1	162	7.34	0.0000000000	0.0000000001
HIST1H4L	393	6.60	0.0000000000	0.0000000001
RP11-767I20.1	146.6	7.57	0.0000000000	0.0000000001
MAGEA6	403.7	-7.48	0.0000000000	0.0000000001
HIST1H2BJ	874.1	5.90	0.0000000000	0.0000000001
DPEP3	814.6	-7.02	0.0000000000	0.0000000001
TPRX1	391.1	-6.89	0.0000000000	0.0000000002
RSPO1	419.6	-6.57	0.0000000000	0.0000000002
STRIP2	3805.8	5.30	0.0000000000	0.0000000002
HIST1H2BE	616.4	6.16	0.0000000000	0.0000000003
XIST	547	6.12	0.0000000000	0.0000000004
RP11-742N3.1	317.9	5.82	0.0000000000	0.0000000005
MAGEA3	344.8	-7.28	0.0000000000	0.0000000005
RP11-313J2.1	441.4	-6.07	0.0000000000	0.0000000007
PTHLH	1351.6	6.89	0.0000000000	0.0000000007
DACT2	136.6	7.10	0.0000000000	0.0000000007
LINC00261	118.2	7.75	0.0000000000	0.0000000007
GP2	115.4	7.72	0.0000000000	0.0000000009
RP11-453F18__B.1	1034.3	-5.29	0.0000000000	0.0000000010
GFAP	609.8	5.98	0.0000000000	0.0000000010
KCNC2	818.5	-5.36	0.0000000000	0.0000000018
HMOX1	1374.1	5.13	0.0000000000	0.0000000019
XAGE5	108.3	7.62	0.0000000000	0.0000000019
CNTNAP4	1104	-6.78	0.0000000000	0.0000000023

BAI3	1655.6	-5.02	0.0000000000	0.0000000025
PCDH8	454	5.80	0.0000000000	0.0000000028
HIST1H2AG	1128.7	5.82	0.0000000000	0.0000000030
LECT1	1973.4	6.67	0.0000000000	0.0000000047
CACNG4	819.9	-5.45	0.0000000000	0.0000000067
CES1	311.8	5.43	0.0000000000	0.0000000072
IGF2BP3	363.6	5.59	0.0000000000	0.0000000076
GLDC	965.1	-5.12	0.0000000000	0.0000000078
SDR42E1	94	7.42	0.0000000000	0.0000000097
KCNQ2	521.9	-5.42	0.0000000000	0.0000000105
HTR1A	842.7	-6.69	0.0000000000	0.0000000110
CTD-2303H24.2	216.9	5.55	0.0000000000	0.0000000124
FOXR2	257.5	-6.34	0.0000000001	0.0000000214
DDX43	247	-6.29	0.0000000001	0.0000000288
CCDC39	261.3	-6.79	0.0000000001	0.0000000335
CCDC172	257.9	5.62	0.0000000001	0.0000000410
NLGN4Y	290.5	-5.76	0.0000000001	0.0000000410
SRD5A2	284.6	5.91	0.0000000001	0.0000000410
KCNH7	260.1	-5.94	0.0000000002	0.0000000490
AC018890.6	108.7	6.45	0.0000000002	0.0000000503
ARL4D	7085.1	4.45	0.0000000002	0.0000000523
DROSHA	2190.6	-4.81	0.0000000002	0.0000000613
HIST1H2AH	401.3	5.65	0.0000000002	0.0000000627
SETD7	2541	4.98	0.0000000002	0.0000000630
HS3ST5	285.3	-5.72	0.0000000003	0.0000000653
SYNDIG1L	222.4	5.43	0.0000000003	0.0000000694
GPR37	585.6	5.60	0.0000000003	0.0000000812
MMP7	126.2	6.20	0.0000000004	0.0000000873

SPAG17	649.8	-5.43	0.0000000004	0.0000000880
RP11-56A10.1	222.8	5.20	0.0000000004	0.0000000913
UGT3A1	602.1	-6.55	0.0000000004	0.0000000978
C1orf132	3349.9	4.65	0.0000000005	0.0000001132
RNF152	2168.9	4.44	0.0000000005	0.0000001226
TFCP2L1	282.9	4.91	0.0000000006	0.0000001451
LSR	267.2	4.98	0.0000000007	0.0000001644
DCT	6038.7	-4.32	0.0000000009	0.0000001889
AC005863.1	363.5	-5.64	0.0000000009	0.0000001889
PAGE5	304.9	-6.59	0.0000000011	0.0000002256
HIST1H4F	354.8	5.21	0.0000000011	0.0000002327
RP11-3N2.13	220.5	-6.06	0.0000000011	0.0000002327
RN7SL5P	3930	4.73	0.0000000013	0.0000002604
NUTM2A-AS1	221	-5.71	0.0000000015	0.0000002982
CABP2	405.3	-6.28	0.0000000017	0.0000003456
FBN3	296.6	-5.28	0.0000000018	0.0000003569
NEGR1	1177.4	-4.53	0.0000000019	0.0000003827
GFRA1	6497	5.36	0.0000000027	0.0000005417

Supplementary Table 2. Comparison of gene expression using RNAseq between pineoblastoma clinical samples PB6 and PB10 with gain of *PDE4DIP*, vs pineoblastoma PB7 with concurrent gain of *PDE4DIP* and homozygous loss of *DROSHA*.

miRNA	PB6	PB10	PB7
hsa-miR-1246	6.438958148	13.8711968	0.495695163
hsa-miR-216b	9.786841364	11.05220801	0.495695163
hsa-miR-182-5p	8.873905455	9.623369416	0.495695163
hsa-miR-130b-3p	7.8928767	8.642484978	0.495695163
hsa-miR-33a-5p	6.357903839	9.050229588	0.495695163
hsa-miR-4284	7.420886575	8.564873515	0.495695163
hsa-miR-513b	8.759155834	4.974988112	0.495695163
hsa-miR-508-3p	8.320033314	1.646162657	0.495695163
hsa-miR-630	5.882887553	7.764075401	0.495695163
hsa-miR-873-5p	6.450221496	7.048977225	0.495695163
hsa-miR-507	7.525285887	0.298658316	0.495695163
hsa-miR-767-5p	5.744161096	6.87835751	0.495695163
hsa-miR-493-3p	6.897845456	7.317503062	0.815575429
hsa-miR-513c-5p	7.976707312	0.298658316	0.815575429
hsa-miR-652-3p	4.801158656	7.465566405	0.815575429
hsa-miR-874-5p	6.039577103	6.937462101	0.815575429
hsa-miR-545-3p	5.68846014	7.088735246	0.815575429
hsa-miR-137	7.386293635	0.298658316	0.815575429
hsa-miR-1206	8.362163012	8.765965634	1.66448284
hsa-miR-135a-5p	3.044394119	8.790250739	1.66448284
hsa-miR-375	3.699329526	8.462829424	1.66448284
hsa-miR-219-5p	5.64990282	6.937462101	1.66448284
hsa-miR-548b-3p	8.172127437	8.578863256	2.195347598
hsa-miR-1290	4.966245873	8.336640446	2.195347598
hsa-miR-105-5p	7.251813755	7.911571752	2.582556003
hsa-miR-425-5p	6.284662185	7.372168569	2.582556003
hsa-miR-124-3p	12.70668379	13.92083572	2.887525271
hsa-miR-488-3p	10.11733141	10.81348461	2.887525271

hsa-miR-210	5.057016965	7.671293372	2.887525271
hsa-miR-216a	7.172827507	8.411595725	3.137503524
hsa-miR-539-5p	6.897845456	7.372168569	3.351910961
hsa-miR-484	5.630230716	6.520265084	3.351910961
hsa-miR-421	8.13442632	9.34881666	3.538538164
hsa-miR-204-5p	9.229900306	7.581426574	3.538538164
hsa-miR-454-3p	6.259460816	7.361417467	3.538538164
hsa-miR-1537	5.086189313	7.455491621	3.538538164
hsa-miR-217	9.079191415	10.26100184	3.703765179
hsa-miR-514a-3p	7.332797428	4.432959407	3.703765179
hsa-miR-582-5p	3.771885579	10.02053554	3.98550043
hsa-miR-597	5.395405605	7.403949364	4.108524457
hsa-miR-181a-3p	6.284662185	8.347577079	4.221877081
hsa-miR-628-5p	6.024585638	8.216357854	4.221877081
hsa-miR-301b	5.549361133	7.475571321	4.326968712
hsa-miR-551a	6.024585638	6.718772592	4.326968712
hsa-miR-135b-5p	13.34802129	7.164906927	4.602290585
hsa-miR-769-5p	9.048187133	9.770961155	4.602290585
hsa-miR-642a-5p	7.77748547	3.949534933	4.602290585
hsa-miR-491-5p	6.404800964	6.980368105	4.602290585
hsa-miR-324-5p	9.775741953	11.11763012	4.683696454
hsa-miR-500a-5p+hsa-miR-501-5p	4.253989266	8.578863256	4.683696454
hsa-miR-196a-5p	5.196921734	8.025028794	4.683696454
hsa-miR-4443	6.404800964	7.87418219	4.760753208
hsa-miR-335-5p	7.03815081	6.558420713	4.760753208
hsa-miR-7-5p	10.67053233	17.69230505	4.833396134
hsa-miR-18a-5p	8.470740208	11.97419946	4.833396134
hsa-miR-577	5.274634403	8.353014462	4.833396134
hsa-miR-194-5p	6.733218743	8.071998581	4.90303827
hsa-miR-34c-5p	9.336439612	9.897421961	4.969472865

hsa-miR-651	5.7977912	9.220692711	4.969472865
hsa-miR-221-3p	4.574101508	8.45779126	4.969472865
hsa-miR-96-5p	9.818550224	10.69418374	5.032982417
hsa-miR-185-5p	5.569855608	7.328585177	5.032982417
hsa-miR-505-3p	5.086189313	8.240075769	5.093391153
hsa-miR-149-5p	8.373996142	9.838479109	5.26190686
hsa-miR-181b-5p+hsa-miR-181d	7.627533884	9.365075712	5.313971371
hsa-miR-532-5p	4.653633311	9.083346354	5.363871925
hsa-miR-590-5p	5.506843099	8.416797528	5.363871925
hsa-miR-885-5p	4.966245873	6.735657559	5.363871925
hsa-miR-551b-3p	12.06460469	14.17264862	5.412442825
hsa-miR-181c-5p	6.778340011	8.745405422	5.412442825
hsa-miR-1180	9.686132803	10.5699695	5.459431619
hsa-miR-151a-3p	7.790772038	8.995597578	5.459431619
hsa-miR-423-3p	5.249066034	7.435128498	5.504938132
hsa-miR-151a-5p	6.568032105	8.821997144	5.591559746
hsa-miR-1260a	6.357903839	7.382840144	5.591559746
hsa-miR-887	6.515699838	6.243745235	5.591559746
hsa-miR-628-3p	6.233619677	7.237353386	5.63314055
hsa-miR-183-5p	11.18255575	10.91357759	5.788163657
hsa-miR-19a-3p	9.715464423	12.2794713	5.824513297
hsa-miR-598	10.51840728	11.3125143	5.859969548
hsa-miR-197-3p	4.489286023	6.356495988	5.961160258
hsa-miR-331-3p	7.186262836	9.307360214	5.993447932
hsa-miR-362-3p	2.9202933	9.070201465	5.993447932
hsa-miR-374b-5p	8.320033314	9.169173402	6.025028794
hsa-miR-320e	7.172827507	8.004445211	6.055716264
hsa-miR-92a-3p	6.733218743	7.997518235	6.115615931
hsa-miR-132-3p	7.530523387	9.253091083	6.173127433
hsa-miR-495	6.167317781	5.185073638	6.173127433

hsa-miR-301a-3p	10.16232838	12.33175391	6.200849575
hsa-miR-379-5p	5.882887553	5.279471296	6.255123015
hsa-miR-106b-5p	11.65995131	12.81579755	6.307246355
hsa-miR-612	5.7977912	5.451870479	6.332707934
hsa-miR-382-5p	6.125981654	4.060047384	6.382321407
hsa-miR-299-3p	6.38145643	7.796429035	6.406502657
hsa-miR-186-5p	8.848716822	9.471492326	6.430117975
hsa-miR-146a-5p	5.027242536	7.990501111	6.430117975
hsa-miR-455-5p	5.762083004	10.19433826	6.476543706
hsa-miR-660-5p	3.454175893	9.838479109	6.476543706
hsa-miR-9-5p	11.79357893	13.82824163	6.499208091
hsa-miR-193b-3p	6.769375049	8.416797528	6.564987801
hsa-miR-518b	5.196921734	6.171727244	6.648321627
hsa-miR-664-3p	9.360408175	9.544674706	6.746178381
hsa-miR-363-3p	6.588414824	6.863195112	6.855491443
hsa-miR-30a-3p	3.971773447	5.410748128	6.872951851
hsa-miR-195-5p	4.934516502	4.589164237	6.940636607
hsa-miR-340-5p	10.49964661	12.28304204	7.097084016
hsa-miR-361-3p	7.988287039	9.682924378	7.111865964
hsa-miR-362-5p	4.653633311	10.20791725	7.12649799
hsa-miR-128	6.787249608	9.793148077	7.169423979
hsa-miR-574-3p	2.63691458	2.485426827	7.329572126
hsa-miR-190a	8.718019065	10.96691636	7.354557947
hsa-miR-296-5p	8.063179335	9.708635594	7.354557947
hsa-miR-30c-5p	6.416333089	8.849217421	7.37920506
hsa-miR-30e-3p	6.648033951	8.887220615	7.462134139
hsa-miR-423-5p	8.933041444	10.6931123	7.529664802
hsa-miR-148b-3p	9.840006812	11.18479478	7.58360934
hsa-miR-19b-3p	10.99913328	13.09554867	7.615004242
hsa-miR-24-3p	6.667466405	7.350585645	7.68580002

hsa-miR-376a-3p	7.718430129	6.399171094	7.695645899
hsa-miR-10b-5p	6.404800964	6.33467553	7.73430317
hsa-miR-107	9.793457	11.77470059	7.826612041
hsa-miR-32-5p	9.458037433	12.23262478	7.844423317
hsa-miR-30e-5p	9.113090983	10.52728151	7.862017377
hsa-miR-127-3p	8.014968933	7.747655933	7.879338185
hsa-miR-136-5p	7.577353292	6.701826258	7.879338185
hsa-miR-361-5p	9.147942623	10.80755618	7.921840937
hsa-miR-106a-5p+hsa-miR-17-5p	12.32005024	13.89972133	7.930205098
hsa-miR-99b-5p	8.338246117	10.82626246	7.938521046
hsa-miR-20a-5p+hsa-miR-20b-5p	12.21224102	13.82860758	7.94673086
hsa-miR-223-3p	7.131651214	8.814101754	7.971256136
hsa-miR-133b	3.158660175	3.215678597	8.003264909
hsa-miR-208b	2.786596362	3.698218478	8.018979052
hsa-miR-133a-5p	0	0.298658316	8.018979052
hsa-miR-222-3p	6.568032105	11.50816617	8.110039536
hsa-miR-93-5p	13.39935003	15.43874313	8.19564288
hsa-miR-140-5p	9.493695363	11.1248063	8.202564648
hsa-miR-486-3p	0	6.42004442	8.269921462
hsa-miR-218-5p	13.34286082	9.208746467	8.340517763
hsa-let-7e-5p	10.39258576	10.75712328	8.413839452
hsa-miR-342-3p	9.793457	11.60258753	8.50608935
hsa-miR-125a-5p	9.316666199	10.27397039	8.560905977
hsa-miR-30b-5p	8.228578221	9.586877309	8.733083132
hsa-miR-503	5.882887553	7.237353386	8.80293635
hsa-miR-499a-5p	5.057016965	7.114575251	8.843292973
hsa-miR-30a-5p	8.148273192	9.340629026	8.86090074
hsa-miR-497-5p	6.13996057	6.312519967	8.9863256
hsa-miR-21-5p	10.83777771	11.27286302	9.265779113
hsa-miR-450a-5p	6.153602696	8.981595822	9.31124879

hsa-miR-365a-3p	7.799605422	8.411595725	9.333468459
hsa-miR-34a-5p	8.286003217	12.25569546	9.352220026
hsa-miR-28-5p	6.297191417	9.32685639	9.352220026
hsa-miR-142-3p	9.533757999	10.59889925	9.416058577
hsa-miR-4286	10.18347411	10.60346872	9.448384785
hsa-miR-148a-3p	10.98859949	10.08410414	9.581596045
hsa-miR-30d-5p	10.66513158	11.93481502	9.589520072
hsa-miR-15b-5p	10.52107037	13.92300986	9.666525827
hsa-miR-145-5p	6.416333089	7.260778432	9.734845078
hsa-miR-27b-3p	8.923000576	9.530347809	9.739611864
hsa-miR-25-3p	13.31917251	16.43338283	9.90550735
hsa-miR-130a-3p	11.42057082	11.2850149	9.928607199
hsa-miR-98	11.82082516	10.195852	9.981809851
hsa-miR-191-5p	13.05244918	13.65025745	9.997786358
hsa-miR-100-5p	8.344251517	9.801142485	10.02338012
hsa-miR-26b-5p	10.10141108	10.17756903	10.05617706
hsa-miR-199a-5p	7.617651119	7.514990142	10.10310418
hsa-miR-144-3p	8.155121802	12.24072277	10.16279412
hsa-let-7f-5p	8.581953751	8.810153867	10.23197714
hsa-miR-374a-5p	11.4322809	12.58345656	10.2520977
hsa-miR-29c-3p	7.283458821	9.481476555	10.40372574
hsa-miR-424-5p	6.914086097	9.930559946	10.49662413
hsa-miR-23b-3p	10.25712851	10.29674417	10.52170839
hsa-miR-26a-5p	10.02288099	10.18673329	10.62435682
hsa-miR-16-5p	9.907656824	13.18276522	10.87527324
hsa-miR-95	6.813524689	8.369248353	10.89353665
hsa-miR-15a-5p	10.83671315	13.41745435	10.91998804
hsa-miR-150-5p	6.369815424	6.33467553	11.05020237
hsa-miR-181a-5p	13.68377539	15.11381785	11.28563918
hsa-let-7i-5p	10.90857276	10.73332044	11.29536717

hsa-miR-378g	6.75141016	8.633576519	11.40803247
hsa-miR-99a-5p	8.451334766	8.401092837	11.41771541
hsa-miR-29a-3p	10.48881423	10.01023436	11.66729342
hsa-miR-199b-5p	7.676098121	7.804389199	11.68467412
hsa-miR-378a-3p+hsa-miR-378i	7.420886575	8.872120779	11.88698446
hsa-miR-22-3p	9.29041058	10.48452083	11.97790238
hsa-let-7c	10.74922418	8.872120779	12.01763315
hsa-let-7d-5p	11.65360197	10.52728151	12.12064937
hsa-let-7g-5p	13.21612743	12.68352102	12.15757115
hsa-miR-126-3p	10.43198852	11.98036811	12.82220234
hsa-miR-125b-5p	9.758939387	9.844297767	13.22864285
hsa-miR-4454	17.57406587	18.03239388	13.56093343
hsa-miR-199a-3p+hsa-miR-199b-3p	10.53100124	11.01320401	13.56110175
hsa-miR-29b-3p	10.49559524	10.82037844	13.72080964
hsa-let-7b-5p	13.12441376	11.10961521	13.90553085
hsa-miR-133a	4.352617299	5.840714991	13.96719473
hsa-miR-23a-3p	10.66753737	11.46671378	14.15091503
hsa-miR-451a	12.27170229	15.56261808	14.41336745
hsa-let-7a-5p	14.99004367	14.83144135	15.11860898
hsa-miR-206	8.566586861	3.3950628	15.15891732
hsa-miR-1	5.249066034	7.524659241	16.14050049

Supplementary Table 3. miRNA quatification in pineoblastoma tumor samples PB6 and PB10 with gain of *PDE4DIP*, vs pineoblastoma PB7 with concurrent gain of *PDE4DIP* and homozygous loss of *DROSHA*. Samples were analyzed using the default quality control measures, and counts were normalized to ligation controls in the Nanostring codesets.

	Drosha-1	Drosha-2	Drosha-3	WT-1	WT-2	WT-3
hsa-miR-571	2.38	2.94	2.86	0.00	0.32	0.00
hsa-miR-487a	0.00	0.03	0.60	1.69	1.90	2.42
hsa-miR-499b-3p	0.00	0.03	0.60	2.01	3.12	2.42
hsa-miR-485-3p	0.00	0.03	0.60	3.26	3.64	2.63
hsa-miR-548l	0.00	0.03	0.60	2.49	2.90	3.40
hsa-miR-195-5p	0.00	0.03	0.60	2.85	2.64	3.51
hsa-miR-885-5p	0.00	0.03	0.60	4.14	4.32	3.51
hsa-miR-1285-5p	2.38	2.20	2.52	3.26	3.12	3.62
hsa-miR-206	0.00	0.03	1.44	2.68	4.13	3.72
hsa-miR-214-3p	0.00	0.03	0.60	3.85	3.64	3.72
hsa-miR-337-5p	0.79	0.03	0.60	3.26	4.96	3.90
hsa-miR-210	1.37	0.03	0.60	3.77	3.12	4.13
hsa-miR-126-3p	0.00	0.03	1.44	4.26	5.18	4.13
hsa-miR-145-5p	0.00	0.03	0.60	5.10	5.71	4.20
hsa-miR-1323	2.11	1.36	0.60	3.77	4.71	4.27
hsa-miR-545-3p	1.79	1.36	0.60	3.93	3.49	4.39
hsa-miR-542-3p	1.37	1.84	0.60	3.49	3.90	4.39
hsa-miR-194-5p	1.37	1.36	0.60	3.26	3.64	4.61
hsa-miR-499a-3p	1.79	0.03	0.60	4.07	4.57	4.85
hsa-miR-10b-5p	0.00	0.63	0.60	5.04	6.34	5.19
hsa-miR-4536-5p	3.49	3.67	2.52	5.10	5.99	5.41
hsa-miR-299-3p	2.60	2.49	1.44	5.46	6.95	5.63
hsa-miR-504	1.79	0.03	0.60	4.38	4.23	5.66
hsa-miR-5196-3p+hsa-miR-6732-3p	0.00	2.20	0.60	6.38	7.98	5.66
hsa-miR-181c-5p	3.25	3.55	2.86	4.85	5.49	5.80
hsa-miR-664-3p	3.49	3.67	3.13	4.96	5.49	5.82
hsa-miR-34c-5p	1.79	0.03	0.60	4.32	4.32	5.86
hsa-miR-592	2.79	2.20	3.13	4.96	5.81	6.01

hsa-miR-216b	0.79	1.36	0.60	5.26	5.88	6.05
hsa-miR-148a-3p	4.31	3.89	3.56	5.48	6.02	6.12
hsa-miR-548h-5p	0.00	0.03	0.60	4.20	5.32	6.16
hsa-miR-362-3p	1.37	1.36	0.60	5.76	5.37	6.58
hsa-miR-483-3p	2.38	0.03	0.60	6.23	7.22	6.64
hsa-miR-26b-5p	3.79	4.31	3.73	6.17	6.37	6.85
hsa-miR-455-3p	3.49	4.07	4.38	5.79	6.97	6.86
hsa-miR-548n	4.25	3.79	3.13	6.39	7.20	6.97
hsa-miR-30c-5p	2.11	1.84	0.60	6.07	6.70	7.00
hsa-miR-365a-3p	3.11	3.79	3.73	6.60	7.08	7.17
hsa-miR-582-5p	3.60	4.07	3.36	6.39	6.61	7.21
hsa-miR-362-5p	4.11	3.55	4.03	6.12	6.32	7.22
hsa-miR-503	4.65	4.76	5.40	6.34	7.09	7.23
hsa-miR-423-3p	4.55	4.86	4.75	6.73	6.97	7.43
hsa-miR-30a-3p	5.00	5.22	4.83	6.89	7.23	7.70
hsa-miR-30a-5p	4.11	5.26	4.28	6.51	6.83	7.80
hsa-miR-342-3p	4.60	4.70	5.29	7.04	7.55	7.96
hsa-miR-95	4.43	5.40	5.04	6.91	7.26	8.01
hsa-miR-92a-3p	5.57	5.63	5.40	7.51	8.12	8.09
hsa-miR-181b-5p+hsa-miR-181d	5.04	5.77	5.84	7.26	7.54	8.17
hsa-miR-423-5p	6.36	6.61	5.91	7.81	8.46	8.18
hsa-miR-548aa	0.00	0.03	0.60	6.51	7.51	8.35
hsa-miR-221-3p	6.22	6.04	5.23	7.81	8.28	8.82
hsa-miR-100-5p	5.43	6.06	5.54	8.09	8.75	8.90
hsa-miR-106b-5p	5.62	6.53	5.63	7.89	8.06	8.92
hsa-miR-361-5p	5.31	6.15	5.68	7.81	7.95	9.01
hsa-miR-148b-3p	6.17	6.43	5.68	8.14	8.25	9.01
hsa-miR-450a-5p	5.28	6.30	5.76	7.98	8.83	9.04
hsa-miR-34a-5p	6.02	6.54	5.50	7.97	8.46	9.35
hsa-miR-26a-5p	6.27	6.76	6.32	9.21	10.13	9.41

hsa-miR-199a-3p+hsa-miR-199b-3p	3.60	4.31	4.67	9.25	9.81	9.70
hsa-miR-21-5p	6.23	6.51	6.32	8.84	9.31	9.92
hsa-miR-92b-3p	8.06	7.75	7.55	9.32	9.83	9.96
hsa-miR-19b-3p	6.13	6.79	6.40	8.60	9.45	9.98
hsa-let-7f-5p	6.60	6.43	5.91	10.08	10.69	10.02
hsa-miR-29b-3p	7.62	7.76	7.38	9.19	9.86	10.13
hsa-miR-20a-5p+hsa-miR-20b-5p	6.81	7.65	7.64	9.02	9.64	10.28
hsa-miR-340-5p	6.50	7.43	7.34	9.27	9.89	10.34
hsa-miR-106a-5p+hsa-miR-17-5p	7.52	8.13	8.35	9.53	10.15	10.48
hsa-miR-99b-5p	7.27	8.03	7.49	9.50	9.67	10.52
hsa-let-7e-5p	7.49	8.11	7.67	9.83	10.12	10.57
hsa-miR-98	7.80	8.14	8.23	10.38	11.11	11.15
hsa-miR-374a-5p	7.23	7.91	8.14	9.85	10.24	11.30
hsa-miR-191-5p	8.06	8.85	8.34	10.24	10.55	11.37
hsa-miR-23a-3p	8.02	8.41	8.18	10.35	10.83	11.51
hsa-miR-25-3p	8.23	8.74	8.79	10.84	11.32	11.85
hsa-miR-16-5p	8.49	9.21	8.94	10.73	11.82	11.95
hsa-miR-181a-5p	9.44	10.00	9.60	11.38	11.85	12.13
hsa-let-7g-5p	8.89	9.27	9.10	11.49	12.04	12.18
hsa-let-7d-5p	8.68	9.04	8.58	11.30	11.75	12.25
hsa-miR-99a-5p	9.30	9.78	9.53	12.09	12.58	12.86
hsa-let-7b-5p	9.81	9.77	9.07	12.41	12.91	12.90
hsa-let-7c	10.39	10.68	10.11	12.72	13.25	13.36
hsa-let-7i-5p	10.99	11.20	10.98	13.27	13.96	13.85
hsa-miR-125b-5p	10.56	11.10	10.28	13.30	13.79	14.22
hsa-let-7a-5p	11.41	11.57	11.33	13.87	14.23	14.60
hsa-miR-9-5p	11.35	11.81	11.15	14.05	14.23	15.35

Supplementary Table 4. miRNA quantification in hNSC lines with induced *DROSHA* aberration vs. control lines. Samples were analyzed using the default quality control measures, and counts were normalized to ligation controls in the Nanostring codesets.

Summary of Clinical Data		
**Age**	All Patients: median (range)<br>Adult: (N=3, 18%)<br>Pediatric: (N=13, 82%)	11 (1–69)<br>45 (37-69)<br>8 (1–17)
**Sex**	Male	4 (29%)
	Female	12 (71%)
**Treatment**	Including Radiation Therapy	16 (100%)
**Overall Survival (years)**	Median (range)	4.0 (1–7)

Supplementary Table 5. Demographic and clinical features of the pineoblastoma patient cohort. For most patients, very limited clinical data were available.

Gene	Confirmed in Samples	Chr:Pos	Tumor Variant Allele Frequency [%]	Coverage	Original Discovery Genome/Exome
ABCA2	PB1-M2	chr9:139906196	48	776	Genome
ABCD2	PB1-M2	chr12:39947853	25	957	Genome
ACOT6	**PB1-M1**, M2, M3	chr14:74086210	21	784	Genome
AGO3	PB8	chr1:36474628	14	389	Exome
ALPK2	PB1-M2	chr18:56204733	24	1078	Genome
ARRB2	PB6	chr17:4619833	14	594	Exome
ARRB2	PB14	chr17:4619841	31	594	Exome
ASTL	PB1-M2	chr2:96789755	27	700	Genome
BDP1	PB1-M2	chr5:70818287	33	574	Genome
C12orf51	PB1-M3	chr12:112600261	5	743	Genome
C5orf42	PB1-M2	chr5:37125473	13	625	Genome
CAPN1	PB6	chr11:64974057	49	579	Exome
CAPN1	PB14	chr11:64974057	43	708	Exome
CHD8	PB8	chr14:21868380	66	524	Exome
COL2A1	**PB1**, PB1-M1, M2, M3	chr12:48371414	22	1766	Genome
CREBBP	PB8	chr16:3824628	61	271	Exome
CSMD2	PB1-M3	chr1:34090148	4	768	Genome
CYP4F2	PB1-M2	chr19:15989696	23	824	Genome
CYSLTR1	PB1	chrX:77529219	16	281	Genome
DCHS2	**PB1-M1**, M2, M3	chr4:155157705	23	736	Genome
DNAH6	PB1-M2	chr2:85024768	27	673	Genome
ELN	**PB1-M1**, M3	chr7:73482996	19	749	Genome
ENPP2	PB1-M2	chr8:120581606	39	1120	Genome
FBN1	PB1	chr15:48760623	15	1515	Genome
GPAT2	PB1	chr2:96688929	38	1641	Genome
GPX5	**PB1**, PB1-M1, M2, M3	chr6:28500155	49	1248	Genome
HBS1L	PB1-M3	chr6:135318704	9	616	Genome
KCNA5	PB1-M2	chr12:5153668	22	951	Genome
KCNN4	PB1	chr19:44273150	30	655	Genome
KRTAP4-4	PB1-M2	chr17:39316742	24	234	Genome
LIG1	PB1	chr19:48626437	3	1442	Genome
LY75-CD302	PB1-M2	chr2:160711423	27	848	Genome
MAP2K7	PB6	chr19:7975205	27	635	Exome
MAP2K7	PB14	chr19:7975205	46	743	Exome
MGLL	**PB1**, PB1-M1, M2, M3	chr3:127500677	46	1507	Genome
MYH3	PB1-M1, M3	chr17:10546195	13	518	Genome
MYO3A	PB1-M2	chr10:26446420	17	1145	Genome
NBPF14	PB1-M1	chr1:148023637	6	1281	Genome
NBPF14	PB6	chr1:148346633	19	586	Exome
NBPF14	PB14	chr1:148346633	17	567	Exome
OR1D5	PB1	chr17:2966549	27	1032	Genome
OR52H1	PB1	chr11:5566168	12	1251	Genome
POU1F1	PB1-M2	chr3:87309234	27	851	Genome
POU6F1	PB1-M2	chr12:51586169	23	991	Genome
PRAMEF11	**PB1**, PB1-M2	chr1:12887174	6	1946	Genome
PTK2B	**PB1**, PB1-M1, M3	chr8:27293840	19	2047	Genome
RASA3	PB1	chr13:114774873	14	1666	Genome
RDBP	**PB1-M1**, M2	chr6:31922412	4	706	Genome
RDH16	PB1-M2	chr12:57351022	19	832	Genome
SALL3	PB1	chr18:76753708	11	1244	Genome
SETD8	PB1	chr12:123879768	9	693	Genome
SLC38A10	PB1-M2	chr17:79226009	9	1010	Genome
SREBF2	PB6	chr22:42276793	48	645	Exome
SREBF2	PB14	chr22:42276793	48	702	Exome

TET1	**PB1-M1**, M2, M3	chr10:70442651	25	584	Genome
TNN	PB14	chr1:175116071	9	794	Exome
TTN	PB1-M2	chr2:179440017	28	882	Genome
TXLNA	PB1-M2	chr1:32655708	22	959	Genome
ZDHHC11	PB1-M1	chr5:833915	23	747	Genome
ZNF292	PB1-M3	chr6:87970549	10	559	Genome
ZNF583	PB1-M2	chr19:56934295	24	1019	Genome
ZNF789	**PB1-M1**, M2, M3	chr7:99085019	22	737	Genome
ZNF8	PB1-M2	chr19:58805617	22	1107	Genome

Supplementary Table 6. Coverage and VAF frequency of confirmed mutations in WES and WGS samples. Variants identified in 8 pineoblastoma samples, where sufficient DNA material was available, were validated on MiSeq platform. Where a mutation was confirmed in several samples from the same patient, coverage and VAF are shown only for the sample in **bold**.
